# Current Awareness on Comparative and Functional Genomics

**DOI:** 10.1002/cfg.425

**Published:** 2005


**Comparative and Functional Genomics**
*Comp Funct Genom* 2005; **6**: 412-430
Published online in Wiley InterScience (www.interscience.wiley.com). **DOI:** 10.1002cfg.425

### Current awareness on comparative and functional genomics

Current Awareness – Use the links online

### 1 Reviews & symposia

Aerssens J, Paulussen ADC. 2005. Pharmacogenomics and acquired long QT syndrome (Review). *Pharmacogenomics J* **6:** (3) 259.
Aksoy S, Berriman M, Hall N, Hattori M, Hide W, Lehane MJ. 2005. A case for a *Glossina* genome project. *Trends Parasitol* **21:** (3) 107.
Anderson L. 2005. Candidate-based proteomics in the search for biomarkers of cardiovascular disease (Review). *J Physiol (Lond)* **563:** (1) 23.
Aslett M, Mooney P, Adlem E, Berriman M, Berry A, Hertz-Fowler C, Ivens AC, Kerhornou A, Parkhill J, Peacock CS *et al*. 2005. Integration of tools and resources for display and analysis of genomic data for protozoan parasites (Review). *Int J Parasitol* **35:** (5) 481.
Banez LL, Srivastava S, Moul JW. 2005. Proteomics in prostate cancer. *Curr Opin Urol* **15:** (3) 151.
Barrett J, Brophy PM, Hamilton JV. 2005. Analysing proteomic data (Review). *Int J Parasitol* **35:** (5) 543.
Barrier M, Mirkes PE. 2005. Proteomics in developmental toxicology. *Reprod Toxicol* **19:** (3) 291.
Battershill JM. 2005. Toxicogenomics: Regulatory perspective on current position. *Hum Exp Toxicol* **24:** (1) 35.
Belli SL, Walker RA, Flowers SA. 2005. Global protein expression analysis in apicomplexan parasites: Current status. *Proteomics* **5:** (4) 918.
Benvenuti S, Arena S, Bardelli A. 2005. Identification of cancer genes by mutational profiling of tumor genomes. *FEBS Lett* **579:** (8) 1884.
Bilello JA. 2005. The agony and ecstasy of "OMIC" technologies in drug development. *Curr Mol Med* **5:** (1) 39.
Biron DG, Moura H, Marche L, Hughes AL, Thomas F. 2005. Towards a new conceptual approach to 'parasitoproteomics'. *Trends Parasitol* **21:** (4) 162.
Brock HW, Fisher CL. 2005. Maintenance of gene expression patterns (Review). *Dev Dyn* **232:** (3) 633.
Burczynski ME, Oestreicher JL, Cahilly MJ, Mounts DP, Whitley MZ, Speicher LA, Trepicchio WL. 2005. Clinical pharmacogenomics and transcriptional profiling in early phase oncology clinical trials. *Curr Mol Med* **5:** (1) 83.
Butcher RA, Schreiber SL. 2005. Using genome-wide transcriptional profiling to elucidate small-molecule mechanism. *Curr Opin Chem Biol* **9:** (1) 25.
Carrillo C, Tulman ER, Delhon G, Lu Z, Carreno A, Vagnozzi A, Kutish GF, Rock DL. 2005. Comparative genomics of foot-and-mouth disease virus (Review). *J Virol* **79:** (10) 6487.
Cavalieri D, De Filippo C. 2005. Bioinformatic methods for integrating whole-genome expression results into cellular networks (Review). *Drug Discov Today* **10:** (10) 727.
Cesareni G, Ceol A, Gavrila C, Palazzi LM, Persico M, Schneider MV. 2005. Comparative interactomics. *FEBS Lett* **579:** (8) 1828.
Chang JC, Hilsenbeck SG, Fuqua SAW. 2005. Genomic approaches in the management and treatment of breast cancer (Review). *Br J Cancer* **92:** (4) 618.
Coppel RL, Vlack CG. 2005. Parasite genomes (Review). *Int J Parasitol* **35:** (5) 465.
Cox B, Kislinger T, Emili A. 2005. Integrating gene and protein expression data: Pattern analysis and profile mining. *Methods* **35:** (3) 303.
Cuperlovic-Culf M, Belacel N, Ouellette RJ. 2005. Determination of tumour marker genes from gene expression data. *Drug Discov Today* **10:** (6) 429.
Delahunty C, Yates JR. 2005. Protein identification using 2D-LC-MS/MS (Review). *Methods* **35:** (3) 248.
Denison C, Kirkpatrick DS, Gygi SP. 2005. Proteomic insights into ubiquitin and ubiquitin-like proteins. *Curr Opin Chem Biol* **9:** (1) 69.
Dracopoli NC. 2005. Development of oncology drug response markers using transcription profiling. *Curr Mol Med* **5:** (1) 103.
Fan HT, Hegde PS. 2005. The transcriptome in blood: Challenges and solutions for robust expression profiling. *Curr Mol Med* **5:** (1) 3.
Fernie AR, Geigenberger P, Stitt M. 2005. Flux an important, but neglected, component of functional genomics. *Curr Opin Plant Biol* **8:** (2) 174.
Fraser CM, Rappuoli R. 2005. Application of microbial genomic science to advanced therapeutics. *Annu Rev Med* **56:** 459.
Gao J, Garulacan LA, Storm SM, Opiteck GJ, Dubaquie Y, Hefta SA, Dambach DM, Dongre AR. 2005. Biomarker discovery in biological fluids. *Methods* **35:** (3) 291.
Garcia BA, Shabanowitz J, Hunt DF. 2005. Analysis of protein phosphorylation by mass spectrometry (Review). *Methods* **35:** (3) 256.
Gingras AC, Aebersold R, Raught B. 2005. Advances in protein complex analysis using mass spectrometry (Review). *J Physiol (Lond)* **563:** (1) 11.
Gismondo MR, De Vecchi E, Drago L. 2004. Use of genomics and proteomics to develop better diagnostic tools for use in infectious diseases. *Rev Med Microbiol* **15:** (3) 103.
Graham DRM, Elliott ST, VanEyk JE. 2005. Broad-based proteomic strategies: A practical guide to proteomics and functional screening (Review). *J Physiol (Lond)* **563:** (1) 1.
Granville CA, Dennis PA. 2005. An overview of lung cancer genomics

In order to keep subscribers up-to-date with the latest developments in their field, this current awareness service is provided by John Wiley & Sons and contains newly-published material on comparative and functional genomics. Each bibliography is divided into 16 sections. 1 Reviews & symposia; 2 General; 3 Large-scale sequencing and mapping; 4 Evolutionary genomics; 5 Comparative genomics; 6 Pathways, gene families and regulons; 7 Pharmacogenomics; 8 EST, cDNA and other clone resources; 9 Functional genomics; 10 Transcriptomics; 11 Proteomics; 12 Protein structural genomics; 13 Metabolomics; 14 Genomic approaches to development; 15 Technological advances; 16 Bioinformatics. Within each section, articles are listed in alphabetical order with respect to author. If, in the preceding period, no publications are located relevant to any one of these headings, that section will be omitted.

Copyright © 2006 John Wiley & Sons, Ltd.

and proteomics. *Am J Respir Cell Mol Biol* **32:** (3) 169.
Hodgson PD, Aich P, Manuja A, Hokamp K, Roche FM, Brinkman FSL, Potter A, Babiuk LA, Griebel PJ. 2005. Effect of stress on viral-bacterial synergy in bovine respiratory disease: Novel mechanisms to regulate inflammation (Conference Review). *Comp Funct Genom* **6:** (4) 244.
Hsu B, Cass L, Williams WV. 2005. Application of transcriptome analysis to clinical pharmacology studies. *Curr Mol Med* **5:** (1) 65.
Hu YF, Kaplow J, He YW. 2005. From traditional biomarkers to transcriptome analysis in drug development. *Curr Mol Med* **5:** (1) 29.
Jenner RG, Young RA. 2005. Insights into host responses against pathogens from transcriptional profiling. *Nat Rev Microbiol* **3:** (4) 281.
John UP, Spangenberg GC. 2005. Xenogenomics: Genomic bioprospecting in indigenous and exotic plants through EST discovery, cDNA microarray-based expression profiling and functional genomics (Conference Review). *Comp Funct Genom* **6:** (4) 230.
Johnson JA, Cavallari LH. 2005. Cardiovascular pharmacogenomics. *Exp Physiol* **90:** (3) 283.
Johnson JM, Edwards S, Shoemaker D, Schadt EE. 2005. Dark matter in the genome: Evidence of widespread transcription detected by microarray tiling experiments (Review). *Trends Genet* **21:** (2) 93.
Johnson RS, Davis MT, Taylor JA, Patterson SD. 2005. Informatics for protein identification by mass spectrometry (Review). *Methods* **35:** (3) 223.
Komatsu S, Tanaka N. 2005. Rice proteome analysis: A step toward functional analysis of the rice genome. *Proteomics* **5:** (4) 938.
Koomen J, Hawke D, Kobayashi R. 2005. Developing an understanding of proteomics: An introduction to biological mass spectrometry. *Cancer Invest* **23:** (1) 47.
Kopec KK, Bozyczko-Coyne D, Williams M. 2005. Target identification and validation in drug discovery: The role of proteomics. *Biochem Pharmacol* **69:** (8) 1133.
Kuruma H, Egawa S, Oh-Ishi M, Kodera Y, Maeda T. 2005. Proteome analysis of prostate cancer (Review). *Prostate Cancer Prostatic Dis* **8:** (1) 14.
LaBaer J, Ramachandran N. 2005. Protein microarrays as tools for functional proteomics. *Curr Opin Chem Biol* **9:** (1) 14.
Lazazzera BA. 2005. Lessons from DNA microarray analysis: The gene expression profile of biofilms. *Curr Opin Microbiol* **8:** (2) 222.
Lee HJ, Yan YL, Marriott G, Corn RM. 2005. Quantitative functional analysis of protein complexes on surfaces (Review). *J Physiol (Lond)* **563:** (1) 61.
Lee NH. 2005. Genomic approaches for reconstructing gene networks (Review). *Pharmacogenomics J* **6:** (3) 245.
Link AJ, Fleischer TC, Weaver CM, Gerbasi VR, Jennings JL. 2005. Purifying protein complexes for mass spectrometry: Applications to protein translation (Review). *Methods* **35:** (3) 274.
MacCross MJ. 2005. Computational analysis of shotgun proteomics data. *Curr Opin Chem Biol* **9:** (1) 88.
Maguire FB, Foy M, Fitzgerald DJ. 2005. Using proteomics to identify potential therapeutic targets in platelets. *Biochem Soc Trans* **33:** (2) 409.
Manasco PK. 2005. Ethical and legal aspects of applied genomic technologies: Practical solutions. *Curr Mol Med* **5:** (1) 23.
Manganaro V, Paratore S, Alessi E, Coffa S, Cavallaro S. 2005. Adding semantics to gene expression profiles: New tools for drug discovery (Review). *Curr Medicinal Chem* **12:** (10) 1149.
Margalit O, Somech R, Amariglio N, Rechavi G. 2005. Microarray-based gene expression profiling of hematologic malignancies: Basic concepts and clinical applications. *Blood Rev* **19:** (4) 223.
McAllister SC, Fruh K, Moses AV. 2005. Functional genomics and the development of pathogenesis-targeted therapies for Kaposi's sarcoma (Review). *Pharmacogenomics J* **6:** (3) 235.
Moritz RL, Skandarajah AR, Ji H, Simpson RJ. 2005. Proteomic analysis of colorectal cancer: Prefractionation strategies using two-dimensional free-flow electrophoresis (Conference Review). *Comp Funct Genom* **6:** (4) 236.
Nishimura T, Ogiwara A, Fujii K, Kawakami T, Kawamura T, Anyouji H, Kato H. 2005. Disease proteomics toward bedside reality. *J Gastroenterol* **40:** (Suppl 16) 7.
O'Rourke NA, Meyer T, Chandy G. 2005. Protein localization studies in the age of 'omics'. *Curr Opin Chem Biol* **9:** (1) 82.
Oksman-Caldentey KM, Saito K. 2005. Integrating genomics and metabolomics for engineering plant metabolic pathways. *Curr Opin Biotechnol* **16:** (2) 174.
Pallen MJ, Beatson SA, Bailey CM. 2005. Bioinformatics, genomics and evolution of non-flagellar type-III secretion systems: A Darwinian perspective. *FEMS Microbiol Rev* **29:** (2) 201.
Park SK, Prolla TA. 2005. Gene expression profiling studies of aging in cardiac and skeletal muscles (Review). *Cardiovasc Res* **66:** (2) 205.
Pelzer S, Vente A, Bechthold A. 2005. Novel natural compounds obtained by genome-based screening and genetic engineering. *Curr Opin Drug Discov Dev* **8:** (2) 228.
Pohl NL. 2005. Functional proteomics for the discovery of carbohydrate-related enzyme activities. *Curr Opin Chem Biol* **9:** (1) 76.
Rasko DA, Altherr MR, Han CS, Ravel J. 2005. Genomics of the *Bacillus cereus* group of organisms. *FEMS Microbiol Rev* **29:** (2) 303.
Saghatelian A, Cravatt BF. 2005. Global strategies to integrate the proteome and metabolome. *Curr Opin Chem Biol* **9:** (1) 62.
Sanoudou D, Vafiadaki E, Arvanitis DA, Kranias E, Kontrogianni-Konstantopoulos A. 2005. Array lessons from the heart: Focus on the genome and transcriptome of cardiomyopathies. *Physiol Genomics* **21:** (2) 131.
Schook LB, Beever JE, Rogers J, Humphray S, Archibald A, Chardon P, Milan D, Rohrer G, Eversole K. 2005. Swine Genome Sequencing Consortium (SGSC): A strategic roadmap for sequencing the pig genome (Conference Review). *Comp Funct Genom* **6:** (4) 251.
Searfoss GH, Ryan TP, Jolly RA. 2005. The role of transcriptome analysis in pre-clinical toxicology. *Curr Mol Med* **5:** (1) 53.
Sharma UC, Pokharel S, Evelo CTA, Maessen JG. 2005. A systematic review of large scale and heterogeneous gene array data in heart failure. *J Mol Cell Cardiol* **38:** (3) 425.
Shin D, Heo YS, Lee KJ, Kim CM, Yoon JM, Lee JI, Hyun YL, Jeon YH, Lee TG, Cho JM *et al*. 2005. Structural chemoproteomics and drug discovery. *Biopolymers* **80:** (2-3) 258.
Sigal S, Ninette A, Rechavi G. 2005. Microarray studies of prognostic stratification and transformation of follicular lymphomas. *Best Pract Res Clin Haematol* **18:** (1) 143.
Simpson DC, Smith RD. 2005. Combining capillary electrophoresis with mass spectrometry for applications in proteomics (Review). *Electrophoresis* **26:** (7-8) 1291.
Simpson KM, Baum J, Good RT, Winzeler EA, Cowman AF, Speed TP. 2005. A comparison of match-only algorithms for the analysis of *Plasmodium falciparum* oligonucleotide arrays (Review). *Int J Parasitol* **35:** (5) 523.
Stoll D, Templin MF, Bachmann J, Joos TO. 2005. Protein microarrays: Applications and future challenges. *Curr Opin Drug Discov Dev* **8:** (2) 239.
Stroncek DF, Burns C, Martin BM, Rossi L, Marincola FM, Panelli MC. 2005. Advancing cancer biotherapy with proteomics (Review). *J Immunother* **28:** (3) 183.
Swanson SK, Washburn MP. 2005. The continuing evolution of shotgun proteomics (Review). *Drug Discov Today* **10:** (10) 719.
Thiede B, Hohenwarter W, Krah A, Mattow J, Schmid M, Schmidt F, Jungblut PR. 2005. Peptide mass fingerprinting. *Methods* **35:** (3) 237.
Thongboonkerd V. 2005. Genomics, proteomics and integrative 'omics' in hypertension research. *Curr Opin Nephrol Hypertens* **14:** (2) 133.
Thongboonkerd V, Malasit P. 2005. Renal and urinary proteomics: Current applications and challenges. *Proteomics* **5:** (4) 1033.
Uttamchandani M, Walsh DP, Yao SQ, Chang YT. 2005. Small molecule microarrays: Recent advances and applications. *Curr Opin Chem Biol* **9:** (1) 4.
Verhelst SHL, Bogyo M. 2005. Chemical proteomics applied to target identification and drug discovery. *Biotechniques* **38:** (2) 175.
Wadlow R, Ramaswamy S. 2005. DNA microarrays in clinical cancer research. *Curr Mol Med* **5:** (1) 111.
Wang XS, Paigen B. 2005. Genome-side search for new genes controlling plasma lipid concentrations in mice and humans. *Curr Opin Lipidol* **16:** (2) 127.
Wick I, Hardiman G. 2005. Biochip platforms as functional genomics tools for drug discovery. *Curr Opin Drug Discov Dev* **8:** (3) 347.
Winslow RL, Cortassa S, Greenstein JL. 2005. Using models of the myocyte for functional interpretation of cardiac proteomic data (Review). *J Physiol (Lond)* **563:** (1) 73.
Worthey EA, Myler PJ. 2005. Protozoan genomes: Gene identification and annotation (Review). *Int J Parasitol* **35:** (5) 495.

Copyright © 2006 John Wiley & Sons, Ltd. 

Wysocki VH, Resing KA, Zhang QF, Cheng GL. 2005. Mass spectrometry of peptides and proteins (Review). *Methods* **35:** (3) 211.

Xiao Z, Prieto D, Conrads TP, Veenstra TD, Issaq HJ. 2005. Proteomic patterns: Their potential for disease diagnosis. *Mol Cell Endocrinol* **230:** (1-2) 95.

Yue L, Reisdorf WC. 2005. Pathway and ontology analysis: Emerging approaches connecting transcriptome data and clinical endpoints. *Curr Mol Med* **5:** (1) 11.

### 3 Large-scale sequencing and mapping

Altermann E, Russell WM, Azcarate-Peril MA, Barrangou R, Buck BL, McAuliffe O, Souther N, Dobson A, Duong T *et al*. 2005. Complete genome sequence of the probiotic lactic acid bacterium *Lactobacillus acidophilus* NCFM. *Proc Natl Acad Sci U S A* **102:** (11) 3906.

Bedell JA, Budiman MA, Nunberg A, Citek RW, Robbins D, Jones J, Flick E, Rohlfing T, Fries J, Bradford K *et al*. 2005. Sorghum genome sequencing by methylation filtration. *PLoS Biol* **3:** (1) e13.

Chiu CH, Tang P, Chu CS, Hu SN, Bao QY, Yu J, Chou YY, Wang HS, Lee YS. 2005. The genome sequence of *Salmonella enterica* serovar Choleraesuis, a highly invasive and resistant zoonotic pathogen. *Nucleic Acids Res* **33:** (5) 1690.

Dean RA, Talbot NJ, Ebbole DJ, Farman ML, Mitchell TK, Orbach MJ, Thon M, Kulkarni R, Xu JR, Pan HQ *et al*. 2005. The genome sequence of the rice blast fungus *Magnaporthe grisea*. *Nature* **434:** (7036) 980.

Eichinger L, Pachebat JA, Glockner G, Rajandream MA, Sucgang R, Berriman M, Song J, Olsen R, Szafranski K, Xu Q *et al*. 2005. The genome of the social amoeba *Dictyostelium discoideum*. *Nature* **435:** (7038) 43.

Fukui T, Atomi H, Kanai T, Matsumi R, Fujiwara S, Imanaka T. 2005. Complete genome sequence of the hyperthermophilic archaeon *Thermococcus kodakaraensis* KOD1 and comparison with *Pyrococcus* genomes. *Genome Res* **15:** (3) 352.

Kwan T, Liu J, DuBow M, Gros P, Pelletier J. 2005. The complete genomes and proteomes of 27 *Staphylococcus aureus* bacteriophages. *Proc Natl Acad Sci U S A* **102:** (14) 5174.

Lee BM, Park YJ, Park DS, Kang HW, Kim JG, Song ES, Park IC, Yoon UH, Hahn JH, Koo BS *et al*. 2005. The genome sequence of *Xanthomonas oryzae* pathovar *oryzae* KACC10331, the bacterial blight pathogen of rice. *Nucleic Acids Res* **33:** (2) 577.

Loftus BJ, Fung E, Roncaglia P, Rowley D, Amedeo P, Bruno D, Vamathevan J, Miranda M, Anderson IJ, Fraser JA *et al*. 2005. The genome of the basidiomycetous yeast and human pathogen *Cryptococcus neoformans*. *Science* **307:** (5713) 1321.

Milosavljevic A, Harris RA, Sodergren EJ, Jackson AR, Kalafus KJ, Hodgson A, Cree A, Dai WL, Csuros M, Zhu BL *et al*. 2005. Pooled genomic indexing of rhesus macaque. *Genome Res* **15:** (2) 292.

Mueller LA, Tanksley SD, Giovannoni JJ, Van Eck J, Stack S, Choi D, Kim BD, Chen M, Cheng Z, Li C *et al*. 2005. The Tomato Sequencing Project, the first cornerstone of the International Solanaceae Project (SOL). *Comp Funct Genom* **6:** (3) 153.

Ruby EG, Urbanowski M, Campbell J, Dunn A, Faini M, Gunsalus R, Lostroh P, Lupp C, McCann J, Millikan D *et al*. 2005. Complete genome sequence of *Vibrio fischeri*: A symbiotic bacterium with pathogenic congeners. *Proc Natl Acad Sci U S A* **102:** (8) 3004.

Stewart JB, Beckenbach AT. 2005. Insect mitochondrial genomics: The complete mitochondrial genome sequence of the meadow spittlebug *Philaenus spumarius* (Hemiptera: Auchenorrhyncha: Cercopoidae). *Genome* **48:** (1) 46.

Vezzi A, Campanaro S, D'Angelo M, Simonato F, Vitulo N, Lauro FM, Cestaro A, Malacrida G, Simionati B, Cannata N *et al*. 2005. Life at depth: *Photobacterium profundum* genome sequence and expression analysis. *Science* **307:** (5714) 1459.

Zhang P, Zhou H, Liang D, Liu YF, Chen YQ, Qu LH. 2005. The complete mitochondrial genome of a tree frog, *Polypedates megacephalus* (Amphibia: Anura: Rhacophoridae), and a novel gene organization in living amphibians. *Gene* **346:** 133.

### 4 Evolutionary genomics

Caetano-Anolles G, Caetano-Anolles D. 2005. Universal sharing patterns in proteomes and evolution of protein fold architecture and life. *J Mol Evol* **60:** (4) 484.

D'Onofrio G, Ghosh TC. 2005. The compositional transition of vertebrate genomes: An analysis of the secondary structure of the proteins encoded by human genes. *Gene* **345:** (1) 27.

Fabre E, Muller H, Therizols P, Lafontaine I, Dujon B, Fairhead C. 2005. Comparative genomics in hemiascomycete yeasts: Evolution of sex, silencing, and subtelomeres. *Mol Biol Evol* **22:** (4) 856.

Foster J, Ganatra M, Kamal I, Ware J, Makarova K, Ivanova N, Bhattacharyya A, Kapatral V, Kumar S, Posfai J *et al*. 2005. The *Wolbachia* genome of *Brugia malayi*: Endosymbiont evolution within a human pathogenic nematode. *PLoS Biol* **3:** (4) e121.

Gill SR, Fouts DE, Archer GL, Mongodin EF, DeBoy RT, Ravel J, Paulsen IT, Kolonay JF, Brinkac L, Beanan M *et al*. 2005. Insights on evolution of virulence and resistance from the complete genome analysis of an early methicillin-resistant *Staphylococcus aureus* strain and a biofilm-producing methicillin-resistant *Staphylococcus epidermidis* strain. *J Bacteriol* **187:** (7) 2426.

Gophna U, Doolittle WF, Charlebois RL. 2005. Weighted genome trees: Refinements and applications. *J Bacteriol* **187:** (4) 1305.

Hsiang T, Baillie DL. 2005. Comparison of the yeast proteome to other fungal genomes to find core fungal genes. *J Mol Evol* **60:** (4) 475.

Jordan IK, Marino-Ramirez L, Koonin EV. 2005. Evolutionary significance of gene expression divergence. *Gene* **345:** (1) 119.

Kunin V, Ahren D, Goldovsky L, Janssen P, Ouzounis CA. 2005. Measuring genome conservation across taxa: Divided strains and united kingdoms. *Nucleic Acids Res* **33:** (2) 616.

Le Gall T, Darlu P, Escobar-Paramo P, Picard B, Denamur E. 2005. Selection-driven transcriptome polymorphism in *Escherichia coli/Shigella* species. *Genome Res* **15:** (2) 260.

Luers AJ, Adams SD, Smalley JV, Campanella JJ. 2005. A phylogenomic study of the genus Alphavirus employing whole genome comparison. *Comp Funct Genom* **6:** (4) 217.

Wagstaff BJ, Begun DJ. 2005. Comparative genomics of accessory gland protein genes in *Drosophila melanogaster* and *D. pseudoobscura*. *Mol Biol Evol* **22:** (4) 818.

Wick LM, Qi WH, Lacher DW, Whittam TS. 2005. Evolution of genomic content in the stepwise emergence of *Escherichia coli* O157:H7. *J Bacteriol* **187:** (5) 1783.

Yu ZG, Zhou LQ, Anh VV, Chu KH, Long SC, Deng JQ. 2005. Phylogeny of prokaryotes and chloroplasts revealed by a simple composition approach on all protein sequences from complete genomes without sequence alignment. *J Mol Evol* **60:** (4) 538.

### 5 Comparative genomics

Ayele M, Haas BJ, Kumar N, Wu H, Xiao YL, Van Aken S, Utterback TR, Wortman JR, White OR, Town CD. 2005. Whole genome shotgun sequencing of *Brassica oleracea* and its application to gene discovery and annotation in *Arabidopsis*. *Genome Res* **15:** (4) 487.

Beckett P, Bancroft I, Trick M. 2005. Computational tools for *Brassica-Arabidopsis* comparative genomics. *Comp Funct Genom* **6:** (3) 147.

Chen S, Zhao SH, McDermott PF, Schroeder CM, White DG, Meng JH. 2005. A DNA microarray for identification of virulence and antimicrobial resistance genes in *Salmonella* serovars and *Escherichia coli*. *Mol Cell Probes* **19:** (3) 195.

Drogemuller C, Wohlke A, Leeb T, Distl O. 2005. A 4 Mb high resolution BAC contig on bovine chromosome 1q12 and comparative analysis with human chromosome 21q22. *Comp Funct Genom* **6:** (4) 194.

Fouts DE, Mongodin EF, Mandrell RE, Miller WG, Rasko DA, Ravel J, Brinkac LM, DeBoy RT, Parker CT, Daugherty SC *et al*. 2005. Major structural differences and novel potential virulence mechanisms from the genomes of multiple *Campylobacter* species. *PLoS Biol* **3:** (1) e15.

Fushan A, Monastyrskaya G, Abaev I, Kostina M, Filyukova O, Pecherskih E, Sverdlov E. 2005. Genome-wide identification and mapping of variable sequences in the genomes of *Burkholderia mallei* and *Burkholderia pseudomallei*. *Res Microbiol* **156:** (2) 278.

Halling SM, Peterson-Burch BD, Bricker BJ, Zuerner RL, Qing Z, Li LL, Kapur V, Alt DP, Olsen SC. 2005. Completion of the genome sequence of *Brucella abortus* and comparison to the highly similar genomes of *Brucella melitensis* and *Brucella suis*. *J Bacteriol* **187:** (8) 2715.

Copyright © 2006 John Wiley & Sons, Ltd. 

Katari MS, Balija V, Wilson RK, Martienssen RA, McCombie WR. 2005. Comparing low coverage random shotgun sequence data from *Brassica oleracea* and *Oryza sativa* genome sequence for their ability to add to the annotation of *Arabidopsis thaliana*. *Genome Res* **15:** (4) 496.

Kauser F, Hussain MA, Ahmed I, Ahmad N, Habeeb A, Khan AA, Ahmed N. 2005. Comparing genomes of *Helicobacter pylori* strains from the high-altitude desert of Ladakh, India. *J Clin Microbiol* **43:** (4) 1538.

Kothapalli S, Nair S, Alokam S, Pang T, Khakhria R, Woodward D, Johnson W, Stocker BAD, Sanderson KE, Liu SL. 2005. Diversity of genome structure in *Salmonella enterica* serovar Typhi populations. *J Bacteriol* **187:** (8) 2638.

Mans BJ, Anantharaman V, Aravind L, Koonin EV. 2004. Comparative genomics, evolution and origins of the nuclear envelope and nuclear pore complex. *Cell Cycle* **3:** (12) 1612.

Margulies EH, Maduro VVB, Thomas PJ, Tomkins JP, Amemiya CT, Luo MZ, Green ED. 2005. Comparative sequencing provides insights about the structure and conservation of marsupial and monotreme genomes. *Proc Natl Acad Sci U S A* **102:** (9) 3354.

Meng BZ, Li CH, Wang GZ, Goszczynski D, Gonsalves D. 2005. Complete genome sequences of two new variants of grapevine rupestris stem pitting-associated virus and comparative analyses. *J Gen Virol* **86:** (5) 1555.

Perez-Brocal V, Latorre A, Gil R, Moya A. 2005. Comparative analysis of two genomic regions among four strains of *Buchnera aphidicola*, primary endosymbiont of aphids. *Gene* **345:** (1) 73.

Yang TJ, Kim J, Lim KB, Kwon SJ, Kim JA, Jin M, Park JY, Lim MH, Kim HI, Kim SH, Lim YP, Park BS. 2005. The Korea *Brassica* Genome Project: A glimpse of the *Brassica* genome based on comparative genome analysis with *Arabidopsis*. *Comp Funct Genom* **6:** (3) 138.

Yao YF, Sturdevant DE, Villaruz A, Xu L, Gao Q, Otto M. 2005. Factors characterizing *Staphylococcus epidermidis* invasiveness determined by comparative genomics. *Infect Immun* **73:** (3) 1856.

Yogeeswaran K, Frary A, York TL, Amenta A, Lesser AH, Nasrallah JB, Tanksley SD, Nasrallah ME. 2005. Comparative genome analyses of *Arabidopsis* spp.: Inferring chromosomal rearrangement events in the evolutionary history of *A. thaliana*. *Genome Res* **15:** (4) 505.

Zhao YF, Ma ZH, Sundin GW. 2005. Comparative genomic analysis of the pPT23A plasmid family of *Pseudomonas syringae*. *J Bacteriol* **187:** (6) 2113.

Zhu HY, Choi HK, Cook DR, Shoemaker RC. 2005. Bridging model and crop legumes through comparative genomics. *Plant Physiol* **137:** (4) 1189.

### 6 Pathways, gene families and regulons

Delalande F, Carapito C, Brizard JP, Brugidou C, Van Dorsselaer A. 2005. Multigenic families and proteomics: Extended protein characterization as a tool for paralog gene identification. *Proteomics* **5:** (2) 450.

Jones AK, Grauso M, Sattelle DB. 2005. The nicotinic acetylcholine receptor gene family of the malaria mosquito, *Anopheles gambiae*. *Genomics* **85:** (2) 176.

Katoh M. 2005. Comparative genomics on Wnt3-Wnt9b gene cluster. *Int J Mol Med* **15:** (4) 743.

Katoh M, Katoh M. 2005. Comparative genomics on Wnt5a and Wnt5b genes. *Int J Mol Med* **15:** (4) 749.

Moita C, Simoes S, Moita LF, Jacinto A, Fernandes P. 2005. The cadherin superfamily in *Anopheles gambiae*: A comparative study with *Drosophila melanogaster*. *Comp Funct Genom* **6:** (4) 204.

### 7 Pharmacogenomics

Abe J, Jibiki T, Noma S, Nakajima T, Saito H, Terai M. 2005. Gene expression profiling of the effect of high-dose intravenous Ig in patients with Kawasaki disease. *J Immunol* **174:** (9) 5837.

Agranoff D, Stich A, Abel P, Krishna S. 2005. Proteomic fingerprinting for the diagnosis of human African trypanosomiasis. *Trends Parasitol* **21:** (4) 154.

Akerman GS, Rosenzweig BA, Domon OE, Tsai CA, Bishop ME, McGarrity LJ, MacGregor JT, Sistare FD, Chen JJ, Morris SM. 2005. Alterations in gene expression profiles and the DNA-damage response in ionizing radiation-exposed TK6 cells. *Environ Mol Mutagen* **45:** (2-3) 188.

Babusiak M, Man P, Sutak R, Petrak J, Vyoral D. 2005. Identification of heme binding protein complexes in murine erythroleukemic cells: Study by a novel two-dimensional native separation - liquid chromatography and electrophoresis. *Proteomics* **5:** (2) 340.

Bertucci F, Finetti P, Rougemont J, Charafe-Jauffret E, Cervera N, Tarpin C, Nguyen C, Xerri L, Houlgatte M, Jacquemier J *et al.* 2005. Gene expression profiling identifies molecular subtypes of inflammatory breast cancer. *Cancer Res* **65:** (6) 2170.

Bhattacharya SK, Rockwood EJ, Smith SD, Bonilha VL, Crabb JS, Kuchtey RW, Robertson NG, Peachey NS, Morton CC, Crabb JW. 2005. Proteomics reveal cochlin deposits associated with glaucomatous trabecular meshwork. *J Biol Chem* **280:** (7) 6080.

Bodzon-Kulakowska A, Bierczynska-Krzysik A, Drabik A, Noga M, Kraj A, Suder P, Silberring J. 2005. Morphinome - Proteome of the nervous system after morphine treatment. *Amino Acids* **28:** (1) 13.

Borlak J, Meier T, Halter R, Spanel R, Spanel-Borowski K. 2005. Epidermal growth factor-induced hepatocellular carcinoma: Gene expression profiles in precursor lesions, early stage and solitary tumours. *Oncogene* **24:** (11) 1809.

Boyd-Kimball D, Sultana R, Poon HF, Lynn BC, Casamenti F, Pepeu G, Klein JB, Butterfield DA. 2005. Proteomic identification of proteins specifically oxidized by intracerebral injection of amyloid β peptide (1-42) into rat brain: Implications for Alzheimer's disease. *Neuroscience* **132:** (2) 313.

Bozinovski S, Cross M, Vlahos R, Jones JE, Hsuu K, Tessier PA, Reynolds EC, Hume DA, Hamilton JA, Geczy CL *et al.* 2005. S100A8 chemotactic protein is abundantly increased, but only a minor contributor to LPS-induced, steroid resistant neutrophilic lung inflammation *in vivo*. *J Proteome Res* **4:** (1) 136.

Braam B, De Roos R, Bluyssen H, Kemmeren P, Holstege F, Joles JA, Koomans H. 2005. Nitric oxide-dependent and nitric oxide-independent transcriptional responses to high shear stress in endothelial cells. *Hypertension* **45:** (4) 672.

Busch A, Michel S, Hoppe C, Driesch D, Claussen U, Von Eggeling F. 2005. Proteome analysis of maternal serum samples for trisomy 21 pregnancies using ProteinChip arrays and bioinformatics. *J Histochem Cytochem* **53:** (3) 341.

Caputo E, Lombardi ML, Luongo V, Moharram R, Tornatore P, Pirozzi G, Guardiola J, Martin BA. 2005. Peptide profiling in epithelial tumor plasma by the emerging proteomic techniques. *J Chromatogr B* **819:** (1) 59.

Centeno BA, Enkemann SA, Coppola D, Huntsman S, Bloom G, Yeatman TJ. 2005. Classification of human tumors using gene expression profiles obtained after microarray analysis of fine-needle aspiration biopsy samples. *Cancer Cytopathol* **105:** (2) 101.

Chandra H, Gupta PK, Sharma K, Mattoo AR, Garg SK, Gade WN, Sirdeshmukh R, Maithal K, Singh Y. 2005. Proteome analysis of mouse macrophages treated with anthrax lethal toxin. *Biochim Biophys Acta* **1747:** (2) 151.

Chang JC, Wooten EC, Tsimelzon A, Hilsenbeck SG, Gutierrez MC, Tham YL, Kalidas M, Elledge R, Mohsin S, Osborne CK *et al.* 2005. Patterns of resistance and incomplete response to docetaxel by gene expression profiling in breast cancer patients. *J Clin Oncol* **23:** (6) 1169.

Chiu ST, Hsieh FJ, Chen SW, Chen CL, Shu HF, Li H. 2005. Clinicopathologic correlation of up-regulated genes identified using cDNA microarray and real-time reverse transcription-PCR in human colorectal cancer. *Cancer Epidemiol Biomarkers Prev* **14:** (2) 437.

Choi YP, Kang S, Hong SH, Xie XH, Cho NH. 2005. Proteomic analysis of progressive factors in uterine cervical cancer. *Proteomics* **5:** (6) 1481.

Chumbalkar VC, Subhashini C, Dhople VM, Sundaram CS, Jagannadham MV, Kumar KN, Srinivas PNBS, Mythili R, Rao MK, Kulkarni MJ *et al.* 2005. Differential protein expression in human gliomas and molecular insights. *Proteomics* **5:** (4) 1167.

Chung IH, Lee SH, Lee KW, Park SH, Cha KY, Kim NS, Yoo HS, Kim YS, Lee S. 2005. Gene expression analysis of cultured amniotic fluid cell with Down syndrome by DNA microarray. *J Korean Med Sci* **20:** (1) 82.

Cobb JP, Mindrinos MN, Miller-Graziano C, Calvano SE, Baker HV, Xiao WZ, Laudanski K, Brownstein BH, Elson CM, Hayden DL *et al.*

Copyright © 2006 John Wiley & Sons, Ltd. 

2005. Application of genome-wide expression analysis to human health and disease. *Proc Natl Acad Sci U S A* **102:** (13) 4801.
Comer JE, Galindo CL, Chopra AK, Peterson JW. 2005. GeneChip analyses of global transcriptional responses of murine macrophages to the lethal toxin of *Bacillus anthracis*. *Infect Immun* **73:** (3) 1879.
Crockett DK, Fillmore GC, Elenitoba-Johnson KSJ, Lim MS. 2005. Analysis of phosphatase and tensin homolog tumor suppressor interacting proteins by *in vitro* and *in silico* proteomics. *Proteomics* **5:** (5) 1250.
D'Arrigo A, Belluco C, Ambrosi A, Digito M, Esposito G, Bertola A, Fabris M, Nofrate V, Mammano E, Leon A *et al*. 2005. Metastatic transcriptional pattern revealed by gene expression profiling in primary colorectal carcinoma. *Int J Cancer* **115:** (2) 256.
Da Costa RMA, Riou L, Paquola A, Menck CFM, Sarasin A. 2005. Transcriptional profiles of unirradiated or UV-irradiated human cells expressing either the cancer-prone XPB/CS allele or the noncancer-prone XPB/TTD allele. *Oncogene* **24:** (8) 1359.
Daikoku T, Tranguch S, Friedman DB, Das SK, Smith DF, Dey SK. 2005. Proteomic analysis identifies immunophilin FK506 binding protein 4 (FKBP52) as a downstream target of Hoxa10 in the periimplantation mouse uterus. *Mol Endocrinol* **19:** (3) 683.
Dasgupta B, Yi YJ, Chen DY, Weber JD, Gutmann DH. 2005. Proteomic analysis reveals hyperactivation of the mammalian target of rapamycin pathway in neurofibromatosis 1-associated human and mouse brain tumors. *Cancer Res* **65:** (7) 2755.
DeGiorgis JA, Jaffe H, Moreira JE, Carlotti CG, Leite JP, Pant HC, Dosemeci A. 2005. Phosphoproteomic analysis of synaptosomes from human cerebral cortex. *J Proteome Res* **4:** (2) 306.
DeSouza L, Diehl G, Rodrigues MJ, Guo JZ, Romaschin AD, Colgan TJ, Siu KWM. 2005. Search for cancer markers from endometrial tissues using differentially labeled tags iTRAQ and cICAT with multidimensional liquid chromatography and tandem mass spectrometry. *J Proteome Res* **4:** (2) 377.
Di Bernardo D, Thompson MJ, Gardner TS, Chobot SE, Eastwood EL, Wojtovich AP, Elliott SJ, Schaus SE, Collins JJ. 2005. Chemogenomic profiling on a genomewide scale using reverse-engineered gene networks. *Nat Biotechnol* **23:** (3) 377.
Dominiczak AF, Graham D, McGride MW, Brain NJR, Lee WK, Charchar FJ, Tomaszewski M, Delles C, Hamilton CA. 2005. Cardiovascular genomics and oxidative stress. *Hypertension* **45:** (4) 636.
Donners MMPC, Verluyten MJ, Bouwman FG, Mariman ECM, Devreese B, Vanrobaeys F, Van Beeumen J, Van den Akker LHJM, Daemen MJAP, Heeneman S. 2005. Proteomic analysis of differential protein expression in human atherosclerotic plaque progression. *J Pathol* **206:** (1) 39.
Drew JE, Rucklidge GJ, Duncan G, Lufty A, Farquharson AJ, Reid MD, Russell WR, Morrice PC, Arthur JR, Duthie GG. 2005. A proteomic approach to identify changes in protein profiles in pre-cancerous colon. *Biochem Biophys Res Commun* **330:** (1) 81.
Drubin D, Smith JS, Liu WL, Zhao W, Chase GA, Clawson GA. 2005. Comparison of cryopreservation and standard needle biopsy for gene expression profiling of human breast cancer specimens. *Breast Cancer Res Treat* **90:** (1) 93.
Duffy PE, Krzych U, Francis S, Fried M. 2005. Malaria vaccines: Using models of immunity and functional genomics tools to accelerate the development of vaccines against *Plasmodium falciparum*. *Vaccine* **23:** (17-18) 2235.
Dupont A, Corseaux D, Dekeyzer O, Drobecq H, Guihot AL, Susen S, Vincentelli A, Amouyel P, Jude B, Pinet F. 2005. The proteome and secretome of human arterial smooth muscle cells. *Proteomics* **5:** (2) 585.
Ebert MPA, Lamer S, Meuer J, Malfertheiner P, Reymond M, Buschmann T, Rocken C, Seibert V. 2005. Identification of the thrombin light chain A as the single best mass for differentiation of gastric cancer patients from individuals with dyspepsia by proteome analysis. *J Proteome Res* **4:** (2) 586.
Ebert MPA, Kruger S, Fogeron ML, Lamer S, Chen J, Pross M, Schulz HU, Lage H, Heim S, Roessner A *et al*. 2005. Overexpression of cathepsin B in gastric cancer identified by proteome analysis. *Proteomics* **5:** (6) 1693.
Ely MR, Nees M, Karsai S, Magele I, Bogumil R, Vorderwulbecke S, Ruess A, Dietz A, Schnolzer M, Bosch FX. 2005. Transcript and proteome analysis reveals reduced expression of calgranulins in head and neck squamous cell carcinoma. *Eur J Cell Biol* **84:** (2-3) 431.
Fan YG, Liu JR, Wang SY, Wang HB, Shi FL, Xiong LH, He W, Peng XX. 2005. Functional proteome of bones in rats with osteoporosis following ovariectomy. *Life Sci* **76:** (25) 2893.
Fannin RD, Auman JT, Bruno ME, Sieber SO, Ward SM, Tucker CJ, Merrick BA, Paules RS. 2005. Differential gene expression profiling in whole blood during acute systemic inflammation in lipopolysaccharide-treated rats. *Physiol Genomics* **21:** (1) 92.
Ferguson SE, Olshen AB, Viale A, Barakat RR, Boyd J. 2005. Stratification of intermediate-risk endometrial cancer patients into groups at high risk or low risk for recurrence based on tumor gene expression profiles. *Clin Cancer Res* **11:** (6) 2252.
Forsberg L, Bjorck E, Hashemi J, Zedenius J, Hoog A, Farnebo LO, Reimers M, Larsson C. 2005. Distinction in gene expression profiles demonstrated in parathyroid adenomas by high-density oligoarray technology. *Eur J Endocrinol* **152:** (3) 459.
Foster JM, Zhang YH, Kumar S, Carlow CKS. 2005. Mining nematode genome data for novel drug targets. *Trends Parasitol* **21:** (3) 101.
Fu YY, Xie C, Yan M, Li Q, Joh JW, Lu C, Mohan C. 2005. The lipopolysaccharide-triggered mesangial transcriptome: Evaluating the role of interferon regulatory factor-1. *Kidney Int* **67:** (4) 1350.
Fuchs D, De Pascual-Teresa S, Rimbach G, Virgili F, Ambra R, Turner R, Daniel H, Wenzel U. 2005. Proteome analysis for identification of target proteins of genistein in primary human endothelial cells stressed with oxidized LDL or homocysteine. *Eur J Nutr* **44:** (2) 95.
Fuchs D, Erhard P, Turner R, Rimbach G, Daniel H, Wenzel U. 2005. Genistein reverses changes of the proteome induced by oxidized-LDL in EA˙hy 926 human endothelial cells. *J Proteome Res* **4:** (2) 369.
Fujii K, Nakano T, Kanazawa M, Akimoto S, Hirano T, Kato H, Nishimura T. 2005. Clinical-scale high-throughput human plasma proteome clinical analysis: Lung adenocarcinoma. *Proteomics* **5:** (4) 1150.
Fujii K, Kondo T, Yokoo H, Yamada T, Iwatsuki K, Hirohashi S. 2005. Proteomic study of human hepatocellular carcinoma using two-dimensional difference gel electrophoresis with saturation cysteine dye. *Proteomics* **5:** (5) 1411.
Galindo CL, Fadl AA, Sha H, Pillai L, Gutierrez C, Chopra AK. 2005. Microarray and proteomics analyses of human intestinal epithelial cells treated with the *Aeromonas hydrophila* cytotoxic enterotoxin. *Infect Immun* **73:** (5) 2628.
Ghobrial IM, McCormick DJ, Kaufmann SH, Leontovich AA, Loegering DA, Dai NT, Krajnik KL, Stenson MJ, Melhem MF, Novak AJ *et al*. 2005. Proteomic analysis of mantle-cell lymphoma by protein microarray. *Blood* **105:** (9) 3722.
Gilks CB, Vanderhyden BC, Zhu S, Van de Rijn M, Longacre TA. 2005. Distinction between serous tumors of low malignant potential and serous carcinomas based on global mRNA expression profiling. *Gynecol Oncol* **96:** (3) 684.
Gillardon F, Steinlein P, Burger E, Hildebrandt T, Gerner C. 2005. Phosphoproteome and transcriptome analysis of the neuronal response to a CDK5 inhibitor. *Proteomics* **5:** (5) 1299.
Goerttler PS, Kreutz C, Donauer J, Faller D, Maiwald T, Marz E, Rumberger B, Sparna T, Schmitt-Graff A, Wilpert J *et al*. 2005. Gene expression profiling in polycythaemia vera: Overexpression of transcription factor NF-E2. *Br J Haematol* **129:** (1) 138.
Gonzalez-Moreno O, Calvo A, Joshi BH, Abasolo I, Leland P, Wang Z, Montuenga L, Puri RK, Green JE. 2005. Gene expression profiling identifies IL-13 receptor $\alpha$2 chain as a therapeutic target in prostate tumor cells overexpressing adrenomedullin. *Int J Cancer* **114:** (6) 870.
Griffiths MJ, Shafi MJ, Popper SJ, Hemingway CA, Kortok MM, Wathen A, Rockett KA, Mott R, Levin M, Newton CR *et al*. 2005. Genomewide analysis of the host response to malaria in Kenyan children. *J Infect Dis* **191:** (10) 1599.
Grus FH, Podust VN, Bruns K, Lackner K, Fu SY, Dalmasso EA, Wirthlin A, Pfeiffer N. 2005. SELDI-TOF-MS ProteinChip Array profiling of tears from patients with dry eye. *Invest Ophthalmol Vis Sci* **46:** (3) 863.
Gunther EC, Stone DJ, Rothberg JM, Gerwien RW. 2005. A quantitative genomic expression analysis platform for multiplexed *in vitro* prediction of drug action. *Pharmacogenomics J* **5:** (2) 126.
Guo JC, Gao HM, Chen J, Zhao P, Cao XD, Li Y, Cheng JS. 2004. Modulation of the gene expression in the protective effects of electroacupuncture against cerebral ischemia: A cDNA microarray study. *Acupunct Electrother Res* **29:** (3-4) 173.
Guteerrez NC, Lopez-Perez R, Hernandez JM, Isidro I, Gonzalez B,

Copyright © 2006 John Wiley & Sons, Ltd. 

Delgado M, Ferminan E, Garcia JL, Vazquez L, Gonzalez M *et al*. 2005. Gene expression profile reveals deregulation of genes with relevant functions in the different subclasses of acute myeloid leukemia. *Leukemia* **19:** (3) 402.
Ha MK, Chung KY, Bang D, Park YK, Lee KH. 2005. Proteomic analysis of the proteins expressed by hydrogen peroxide treated culture human dermal microvascular endothelial cells. *Proteomics* **5:** (6) 1507.
Haas DW. 2005. Pharmacogenomics and HIV therapeutics. *J Infect Dis* **191:** (9) 1397.
Hanlon PR, Zheng WC, Ko AY, Jefcoate CR. 2005. Identification of novel TCDD-regulated genes by microarray analysis. *Toxicol Appl Pharmacol* **202:** (3) 215.
Hanna J, Fitchett J, Rowe T, Daniels M, Heller M, Gonen-Gross T, Manaster E, Cho SY, LaBarre MJ, Mandelboim O. 2005. Proteomic analysis of human natural killer cells: Insights on new potential NK immune functions. *Mol Immunol* **42:** (4) 425.
Hardt M, Thomas LR, Dixon SE, Newport G, Agabian N, Prakobphol A, Hall SC, Witkowska HE, Fisher SJ. 2005. Toward defining the human parotid gland salivary proteome and peptidome: Identification and characterization using 2D SDS-PAGE, ultrafiltration, HPLC, and mass spectrometry. *Biochemistry* **44:** (8) 2885.
Hardwick JS, Yang Y, Zhang CS, Shi B, McFall R, Koury EJ, Hill SL, Dai HY, Wasserman R, Phillips RL *et al*. 2005. Identification of biomarkers for tumor endothelial cell proliferation through gene expression profiling. *Mol Cancer Ther* **4:** (3) 413.
Hartmann LC, Lu KH, Linette GP, Cliby WA, Kalli KR, Gershenson D, Bast RC, Stec J, Iartchouk N, Smith DI *et al*. 2005. Gene expression profiles predict early relapse in ovarian cancer after platinum-paclitaxel chemotherapy. *Clin Cancer Res* **11:** (6) 2749.
Hathout Y, Flippin J, Fan CG, Liu PH, Csaky K. 2005. Metablic labeling of human primary retinal pigment epithelial cells for accurate comparative proteomics. *J Proteome Res* **4:** (2) 620.
Hauptschein RS, Sloan KE, Torella C, Moezzifard R, Giel-Moloney M, Zehetmeier C, Unger C, Llag LL, Jay DG. 2005. Functional proteomic screen identifies a modulating role for CD44 in death receptor-mediated apoptosis. *Cancer Res* **65:** (5) 1887.
Hayashi E, Kuramitsu Y, Okada F, Fujimoto M, Zhang XL, Kobayashi M, Lizuka N, Ueyama Y, Nakamura K. 2005. Proteomic profiling for cancer progression: Differential display analysis for the expression of intracellular proteins between regressive and progressive cancer cell lines. *Proteomics* **5:** (4) 1024.
Hayashi J, Stoyanova R, Seeger C. 2005. The transcriptome of HCV replicon expressing cell lines in the presence of α interferon. *Virology* **335:** (2) 264.
Heidenblad M, Lindgren D, Veltman JA, Jonson T, Mahlamaki EH, Gorunova L, Van Kessel AG, Schoenmakers EFPM, Hoglund M. 2005. Microarray analyses reveal strong influence of DNA copy number alterations on the transcriptional patterns in pancreatic cancer: Implications for the interpretation of genomic implications. *Oncogene* **24:** (10) 1794.
Heike Y, Hosokawa M, Osumi S, Fujii D, Aogi K, Takigawa N, Ida M, Tajiri H, Eguchi K, Shiwa M *et al*. 2005. Identification of serum proteins related to adverse effects induced by docetaxel infusion from protein expression profiles of serum using SELDI ProteinChip system. *Anticancer Res* **25:** (2B) 1197.
Hensbergen PJ, Alewijnse AE, Kempenaar J, Van der Schors RC, Balog CIA, Deelder AM, Beumer G, Ponec M, Tensen CP. 2005. Proteomic profiling identifies an UV-induced activation of cofilin-1 and destrin in human epidermis. *J Investig Dermatol* **124:** (4) 818.
Hewitt SC, Collins J, Grissom S, Deroo B, Korach KS. 2005. Global uterine genomics *in vivo*: Microarray evaluation of the estrogen receptor α-growth factor cross-talk mechanism. *Mol Endocrinol* **19:** (3) 657.
Hill EW, O'Gorman GM, Agaba M, Gibson JP, Hanotte O, Kemp SJ, Naessens J, Coussens PM, MacHugh DE. 2005. Understanding bovine trypanosomiasis and trypanotolerance: The promise of functional genomics. *Vet Immunol Immunopathol* **105:** (3-4) 247.
Hittel DS, Hathout Y, Hoffman EP, Houmard JA. 2005. Proteome analysis of skeletal muscle from obese and morbidly obese women. *Diabetes* **54:** (5) 1283.
Hoefnagel JJ, Dijkman R, Basso K, Jansen PM, Hallermann C, Willemze R, Tensen CP, Vermeer MH. 2005. Distinct types of primary cutaneous large B-cell lymphoma identified by gene expression profiling. *Blood* **105:** (9) 3671.
Hoelzinger DB, Mariani L, Weis J, Woyke T, Berens TJ, McDonough WS, Sloan A, Coons SW, Berens ME. 2005. Gene expression profile of glioblastoma multiforme invasive phenotype points to new therapeutic targets. *Neoplasia* **7:** (1) 7.
Hollingshead D, Lewis DA, Mirnics K. 2005. Platform influence on DNA microarray data in postmortem brain research. *Neurobiol Dis* **18:** (3) 649.
Horcajadas JA, Riesewijk A, Polman J, Van Os R, Pellicer A, Mosselman S, Simon C. 2005. Effect of controlled ovarian hyperstimulation in IVF on endometrial gene expression profiles. *Mol Hum Reprod* **11:** (3) 195.
Hu N, Wang CY, Hu Y, Yang HH, Giffen C, Tang ZZ, Han XY, Goldstein AM, Emmert-Buck MR, Buetow KH *et al*. 2005. Genome-wide association study in esophageal cancer using GeneChip mapping 10K array. *Cancer Res* **65:** (7) 2542.
Hu S, Xie YM, Ramachandran P, Loo RRO, Li Y, Loo JA, Wong DT. 2005. Large-scale identification of proteins in human salivary proteome by liquid chromatography/mass spectrometry and two-dimensional gel electrophoresis-mass spectrometry. *Proteomics* **5:** (6) 1714.
Huang CM, Shi ZK, De Silva TS, Yamamoto M, Van Kampen KR, Elmets CA, Tang DCC. 2005. A differential proteome in tumors suppressed by an adenovirus-based skin patch vaccine encoding human carcinoembryonic antigen. *Proteomics* **5:** (4) 1013.
Huber C, Thielen C, Seeger H, Schwarz P, Montrasio F, Wilson MR, Heinen E, Fu YX, Miele G, Aguzzi A. 2005. Lymphotoxin-β receptor-dependent genes in lymph node and follicular dendritic cell transcriptomes. *J Immunol* **174:** (9) 5526.
Hubner N, Wallace CA, Zimdahl H, Petretto E, Schulz H, Maciver F, Mueller M, Hummel O, Monti J, Zidek V *et al*. 2005. Integrated transcriptional profiling and linkage analysis for identification of genes underlying disease. *Nat Genet* **37:** (3) 243.
Inkinen K, Lahesmaa R, Brandt A, Katajamaa M, Halme L, Hockerstedt K, Lautenschlager I. 2005. DNA microarray-based gene expression profiles of cytomegalovirus infection and acute rejection in liver transplants. *Transplant Proc* **37:** (2) 1227.
Inzitari R, Cabras T, Onnis G, Olmi C, Mastinu A, Sanna MT, Pellegrini MG, Castagnola M, Messana I. 2005. Different isoforms and post-translational modifications of human salivary acidic proline-rich proteins. *Proteomics* **5:** (3) 805.
Ise R, Han DH, Takahashi Y, Terasaka S, Inoue A, Tanji M, Kiyama R. 2005. Expression profiling of the estrogen responsive genes in response to phytoestrogens using a customized DNA microarray. *FEBS Lett* **579:** (7) 1732.
Iwadate Y, Sakaida T, Saegusa T, Hiwasa T, Takiguchi M, Fujimoto S, Yamaura A. 2005. Proteome-based identification of molecular markers predicting chemosensitivity to each category of anticancer agents in human gliomas. *Int J Oncol* **26:** (4) 993.
Jacobs JM, Yang XH, Luft BJ, Dunn JJ, Camp DG, Smith RD. 2005. Proteomic analysis of Lyme disease: Global protein comparison of three strains of *Borrelia burgdorferi*. *Proteomics* **5:** (5) 1446.
James ER, Fresco VM, Robertson LL. 2005. Glucocorticoid-induced changes in the global gene expression of lens epithelial cells. *J Ocul Pharmacol Ther* **21:** (1) 11.
Jiang PZ, Gan M, Huang H, Shen XM, Wang S, Yao KT. 2005. Proteome analysis of antiproliferative mechanism of 12-*O*-tetradecanoylphorbol 13-acetate on cultured nasopharyngeal carcinoma CNE2 cells. *J Proteome Res* **4:** (2) 599.
Jin JH, Meredith GE, Chen L, Zhou Y, Xu J, Shie FS, Lockhart P, Zhang J. 2005. Quantitative proteomic analysis of mitochondrial proteins: Relevance to Lewy body formation and Parkinson's disease. *Mol Brain Res* **134:** (1) 119.
Jin WH, Dai J, Li SJ, Xia QC, Zou HF, Zeng R. 2005. Human plasma proteome analysis by multidimensional chromatography prefractionation and linear ion trap mass spectrometry identification. *J Proteome Res* **4:** (2) 613.
Jones SB, DePrimo SE, Whitfield ML, Brooks JD. 2005. Resveratrol-induced gene expression profiles in human prostate cancer cells. *Cancer Epidemiol Biomarkers Prev* **14:** (3) 596.
Junker H, Spate K, Suofu Y, Walther R, Schwarz G, Kammer W, Nordheim A, Walker LC, Runge U, Kessler C *et al*. 2005. Proteomic identification of the involvement of the mitochondrial Rieske protein in epilepsy. *Epilepsia* **46:** (3) 339.
Kalish JA, Willis DJ, Li C, Link JJ, Deutsch ER, Contreras MA, Quist WC, Logerfo FW. 2004. Temporal genomics of vein bypass grafting

Copyright © 2006 John Wiley & Sons, Ltd. 

through oligonucleotide microarray analysis. *J Vasc Surg* **39:** (3) 645.
Kang D, Nam H, Kim YS, Moon MH. 2005. Dual-purpose sample trap for on-line strong cation-exchange chromatography reversed-phase liquid chromatography tandem mass spectrometry for shotgun proteomics - Application to the human Jurkat T-cell proteome. *J Chromatogr A* **1070:** (1-2) 193.
Kannangai R, Diehl AM, Sicklick J, Rojkind M, Thomas D, Torbenson M. 2005. Hepatic angiomyolipoma and hepatic stellate cells share a similar gene expression profile. *Hum Pathol* **36:** (4) 341.
Kano M, Tsutsumi S, Kawahara N, Wang Y, Mukasa A, Kirino T, Aburatani H. 2005. A meta-clustering analysis indicates distinct pattern alteration between two series of gene expression profiles for induced ischemic tolerance in rats. *Physiol Genomics* **21:** (2) 274.
Karlsson H, Leanderson P, Tagesson C, Lindahl M. 2005. Lipoproteomics II: Mapping of proteins in high-density lipoprotein using two-dimensional gel electrophoresis and mass spectrometry. *Proteomics* **5:** (5) 1431.
Karsan A, Blonder J, Law J, Yaquian E, Lucas DA, Conrads TP, Veenstra T. 2005. Proteomic analysis of lipid microdomains from lipopolysaccharide-activated human endothelial cells. *J Proteome Res* **4:** (2) 349.
Kim SY, Chudapongse N, Lee SM, Levin MC, Oh JT, Park HJ, Ho IK. 2005. Proteomic analysis of phosphotyrosyl proteins in morphine-dependent rat brains. *Mol Brain Res* **133:** (1) 58.
Knoll KE, Pietrusz JL, Liang MY. 2005. Tissue-specific transcriptome responses in rats with early streptozotocin-induced diabetes. *Physiol Genomics* **21:** (2) 222.
Kong SW, Bodyak N, Yue P, Liu ZL, Brown J, Izumo S, Kang PM. 2005. Genetic expression profiles during physiological and pathological cardiac hypertrophy and heart failure in rats. *Physiol Genomics* **21:** (1) 34.
Kume E, Aruga C, Ishizuka Y, Takahashi K, Miwa S, Roh M, Fujimura H, Toriumi W, Kitamura K, Doi K. 2005. Gene expression profiling in streptozotocin treated mouse liver using DNA microarray. *Exp Toxicol Pathol* **56:** (4-5) 235.
Kume E, Aruga C, Takahashi K, Miwa S, Dekura E, Itoh M, Ishizuka Y, Fujimura H, Toriumi W, Doi K. 2005. Morphological and gene expression analysis in mouse primary cultured hepatocytes exposed to streptozotocin. *Exp Toxicol Pathol* **56:** (4-5) 245.
Kung C, Kenski DM, Dickerson SH, Howson RW, Kuyper F, Madhani HD, Shokat KM. 2005. Chemical genomic profiling to identify intracellular targets of a multiplex kinase inhibitor. *Proc Natl Acad Sci U S A* **102:** (10) 3587.
Kuo CC, Kuo CW, Liang CM, Liang SM. 2005. A transcriptomic and proteomic analysis of the effect of CpG-ODN on human THP-1 monocytic leukemia cells. *Proteomics* **5:** (4) 894.
Kuruma H, Egawa S, Oh-Ishi M, Kodera Y, Satoh M, Chen WG, Okusa H, Matsumoto K, Maeda T, Baba S. 2005. High molecular mass proteome of androgen-independent prostate cancer. *Proteomics* **5:** (4) 1097.
Lee CH, Lum JHK, Cheung BPY, Wong MS, Butt YKC, Tam MF, Chan WY, Chow C, Hui PK, Kwok FSL *et al*. 2005. Identification of the heterogeneous nuclear ribonucleoprotein A2/B1 as the antigen for the gastrointestinal cancer specific monoclonal antibody MG7. *Proteomics* **5:** (4) 1160.
Lee SH, Lee DY, Son WK, Joo WA, Kim CW. 2005. Proteomic characterization of rat liver exposed to 2,3,7,8-tetrachlorobenzo-*p* dioxin. *J Proteome Res* **4:** (2) 335.
Leung D, Du W, Hardouin C, Cheng H, Hwang I, Cravatt BF, Boger DL. 2005. Discovery of an exceptionally potent and selective class of fatty acid amide hydrolase inhibitors enlisting proteome-wide selectivity screening: Concurrent optimization of enzyme inhibitor potency and selectivity. *Bioorg Med Chem Lett* **15:** (5) 1423.
Li C, Tan YX, Zhou H, Ding SJ, Li SJ, Ma DJ, Man XB, Hong Y, Zhang L, Li L *et al*. 2005. Proteomic analysis of hepatitis B virus-associated hepatocellular carcinoma: Identification of potential tumor markers. *Proteomics* **5:** (4) 1125.
Li QZ, Li P, Garcia GE, Johnson RJ, Feng LL. 2005. Genomic profiling of neutrophil transcripts in Asian Qigong practitioners: A pilot study in gene regulation by mind-body interaction. *J Altern Complement Med* **11:** (1) 29.
Li YW, Hong X, Hussain M, Sarkar SH, Li R, Sarkar FH. 2005. Gene expression profiling revealed novel molecular targets of docetaxel and estramustine combination treatment in prostate cancer cells. *Mol Cancer Ther* **4:** (3) 389.
Lin BY, White JT, Lu W, Xie T, Utleg AG, Yan XW, Yi EC, Shannon P, Khrebtukova I, Lange PH *et al*. 2005. Evidence for the presence of disease-perturbed networks in prostate cancer cells by genomic and proteomic analyses: A systems approach to disease. *Cancer Res* **65:** (8) 3081.
Liu T, Qian WJ, Chen WNU, Jacobs JM, Moore RJ, Anderson DJ, Gritsenko MA, Monroe ME, Thrall BD, Camp DG *et al*. 2005. Improved proteome coverage by using high efficiency cysteinyl peptide enrichment: The human mammary epithelial cell proteome. *Proteomics* **5:** (5) 1263.
Liu WW, Guan M, Wu DL, Zhang YF, Wu Z, Xu M, Lu Y. 2005. Using tree analysis pattern and SELDI-TOF-MS to discriminate transitional cell carcinoma of the bladder cancer from noncancer patients. *Eur Urol* **47:** (4) 456.
Lovell MA, Xiong SL, Markesbery WR, Lynn BC. 2005. Quantitative proteomic analysis of mitochondria from primary neuron cultures treated with amyloid β peptide. *Neurochem Res* **30:** (1) 113.
Loyet KM, Ouyang WJ, Eaton DL, Stults JT. 2005. Proteomic profiling of surface proteins on Th1 and Th2 cells. *J Proteome Res* **4:** (2) 400.
Luo W, Fan WH, Xie H, Jing LC, Ricicki E, Vouros P, Zhao LP, Zarbl H. 2005. Phenotypic anchoring of global gene expression profiles induced by *N*-hydroxy-4-acetylaminobiphenyl and benzo[*a*]pyrene diol epoxide reveals correlations between expression profiles and mechanism of toxicity. *Chem Res Toxicol* **18:** (4) 619.
Luo XP, Ding L, Xu JX, Williams RS, Chegini N. 2005. Leiomyoma and myometrial gene expression profiles and their responses to gonadotropin-releasing hormone analog therapy. *Endocrinology* **146:** (3) 1074.
Luo XP, Ding L, Xu JX, Chegini N. 2005. Gene expression profiling of leiomyoma and myometrial smooth muscle cells in response to transforming growth factor-β. *Endocrinology* **146:** (3) 1097.
Magni F, Sarto C, Valsecchi C, Casellato S, Bogetto SF, Bosari S, Di Fonzo A, Perego RA, Corizzato M, Doro G *et al*. 2005. Expanding the proteome two-dimensional gel electrophoresis reference map of human and renal cortex by peptide mass fingerprinting. *Proteomics* **5:** (3) 816.
Margulies KB, Matiwala S, Cornejo C, Olsen H, Craven WA, Bednarik D. 2005. Mixed messages - Transcription patterns in failing and recovering human myocardium. *Circ Res* **96:** (5) 592.
McMurry J, Sbai H, Gennaro ML, Carter EJ, Martin W, De Groot AS. 2005. Analyzing *Mycobacterium tuberculosis* proteomes for candidate vaccine epitopes. *Tuberculosis* **85:** (1-2) 95.
Michel C, Roberts RA, Desdouets C, Isaacs KR, Boitier E. 2005. Characterization of an acute molecular marker of nongenotoxic rodent hepatocarcinogenesis by gene expression profiling in a long term clofibric acid study. *Chem Res Toxicol* **18:** (4) 611.
Mini R, Figura N, D'Ambrosio C, Braconi D, Bernardini G, Di Simplicio F, Lenzi C, Nuti R, Trabalzini L, Martelli P *et al*. 2005. *Helicobacter pylori* immunoproteomes in case reports of rosacea and chronic urticaria. *Proteomics* **5:** (3) 777.
Morand JPF, Macri J, Adeli K. 2005. Proteomic profiling of hepatic endoplasmic reticulum-associated proteins in an animal model of insulin resistance and metabolic dyslipidemia. *J Biol Chem* **280:** (18) 17626.
Morgan KT, Jayyosi Z, Hower MA, Pino MV, Connolly TM, Kotlenga K, Lin JY, Wang M, Schmidts HL, Bonnefoi MS *et al*. 2005. The hepatic transcriptome as a window on whole-body physiology and pathophysiology. *Toxicol Pathol* **33:** (1) 136.
Motoori M, Takemasa I, Yano M, Saito S, Miyata H, Takiguchi S, Fujiwara Y, Yasuda T, Doki Y, Kurokawa Y *et al*. 2005. Prediction of recurrence in advanced gastric cancer patients after curative resection by gene expression profiling. *Int J Cancer* **114:** (6) 963.
Muniz EZ, Bruges JM, Vaca FB. 2005. Early cancer diagnosis through proteomics of serum: Fiction or fact? (Spanish, English Abstract). *Med Clin (Barc)* **124:** (5) 181.
Nabetani T, Tabuse Y, Tsugita A, Shoda J. 2005. Proteomic analysis of livers of patients with primary hepatolithiasis. *Proteomics* **5:** (4) 1043.
Nabi G, N'Dow J, Hasan TS, Booth IR, Cash P. 2005. Proteomic analysis of urine in patients with intestinal segments transposed into the urinary tract. *Proteomics* **5:** (6) 1729.
Nakahara Y, Shiraishi T, Okamoto H, Mineta T, Oishi T, Sasaki K, Tabuchi K. 2004. Detrended fluctuation analysis of genome-wide copy number profiles of glioblastomas using array-based comparative

Copyright © 2006 John Wiley & Sons, Ltd. 

genomic hybridization. *Neuro-Oncology* **6:** (4) 281.

Nakanishi F, Ohkawa K, Ishida H, Hosui A, Sato A, Hiramatsu N, Ueda K, Takehara T, Kasahara A, Sasaki Y *et al*. 2005. Alteration in gene expression profile by full-length hepatitis B virus genome. *Intervirology* **48:** (2-3) 77.

Nakatsu N, Yoshida Y, Yamazaki K, Nakamura T, Dan SG, Fukui Y, Yamori T. 2005. Chemosensitivity profile of cancer cell lines and identification of genes determining chemosensitivity by an integrated bioinformatical approach using cDNA arrays. *Mol Cancer Ther* **4:** (3) 399.

Nazarian J, Bouri K, Hoffman EP. 2005. Intracellular expression profiling by laser capture microdissection: Three novel components of the neuromuscular junction. *Physiol Genomics* **21:** (1) 70.

Nelsestuen GL, Martinez MB, Hertz MI, Savik K, Wendt CH. 2005. Proteomic identification of human neutrophil α-defensins in chronic lung allograft rejection. *Proteomics* **5:** (6) 1705.

Nigro JM, Misra A, Zhang L, Smirnov I, Colman H, Griffin C, Ozburn N, Chen MG, Pan E, Koul D *et al*. 2005. Integrated array-comparative genomic hybridization and expression array profiles identify clinically relevant molecular subtypes of glioblastoma. *Cancer Res* **65:** (5) 1678.

Nosho K, Yamamoto H, Adachi Y, Endo T, Hinoda Y, Imai K. 2005. Gene expression profiling of colorectal adenomas and early invasive carcinomas by cDNA array analysis. *Br J Cancer* **92:** (7) 1193.

Ohira M, Oba S, Nakamura Y, Isogai E, Kaneko S, Nakagawa A, Hirata T, Kubo H, Goto T, Yamada S *et al*. 2005. Expression profiling using a tumor-specific cDNA microarray predicts the prognosis of intermediate risk neuroblastomas. *Cancer Cell* **7:** (4) 337.

Ohta K, Kikuchi T, Miyahara T, Yoshimura N. 2005. DNA microarray analysis of gene expression in iris and ciliary body of rat eyes with endotoxin-induced uveitis. *Exp Eye Res* **80:** (3) 401.

Ozyildirim AM, Wistow GJ, Gao J, Wang JH, Dickinson DP, Frierson HF, Laurie GW. 2005. The lacrimal gland transcriptome is an unusually rich source of rare and poorly characterized gene transcripts. *Invest Ophthalmol Vis Sci* **46:** (5) 1572.

Paron I, D'Ambrosio C, Scaloni A, Berlingieri MT, Pallante PL, Fusco A, Bivi N, Tell G, Damante G. 2005. A differential proteomic approach to identify proteins associated with thyroid cell transformation. *J Mol Endocrinol* **34:** (1) 199.

Patino WD, Mian OY, Kang JG, Matoba S, Bartlett LD, Holbrook B, Trout HH, Kozloff L, Hwang PM. 2005. Circulating transcriptome reveals markers of atherosclerosis. *Proc Natl Acad Sci U S A* **102:** (9) 3423.

Pawlowska Z, Baranska P, Jerczynska H, Koziolkiewicz W, Cierniewski CS. 2005. Heat shock proteins and other components of cellular machinery for protein synthesis are up-regulated in vascular endothelial cell growth factor-activated human endothelial cells. *Proteomics* **5:** (5) 1217.

Pereira SR, Faca VM, Gomes GG, Chammas R, Fontes AM, Covas DT, Greene LJ. 2005. Changes in the proteomic profile during differentiation and maturation of human monocyte-derived dendritic cells stimulated with granulocyte macrophage colony stimulating factor/interleukin-4 and lipopolysaccharide. *Proteomics* **5:** (5) 1186.

Perluigi M, Poon HF, Hensley K, Pierce WM, Klein JB, Calabrese V, De Marco C, Butterfield DA. 2005. Proteomic analysis of 4-hydroxy-2-nonenal-modified proteins in G93A-SOD1 transgenic mice - A model of familial amyotrophic lateral sclerosis. *Free Radic Biol Med* **38:** (7) 960.

Permyakov SE, Pershikova IV, Zhadan AP, Goers J, Bakunts AG, Uversky VN, Berliner LJ, Permyakov EA. 2005. Conversion of human α-lactalbumin to an apo-like state in the complexes with basic poly-amino acids: Toward understanding of the molecular mechanism of antitumor action of HAMLET. *J Proteome Res* **4:** (2) 564.

Piubelli C, Cecconi D, Astner H, Caldara F, Tessari M, Carboni L, Hamdan M, Righetti PG, Domenici E. 2005. Proteomic changes in rat serum, polymorphonuclear and mononuclear leukocytes after chronic nicotine administration. *Proteomics* **5:** (5) 1382.

Pocernich CB, Poon HF, Boyd-Kimball D, Lynn BC, Nath A, Klein JB, Butterfield DA. 2005. Proteomic analysis of oxidatively modified proteins induced by the mitochondrial toxin 3-nitropropionic acid in human astrocytes expressing the HIV protein. *Mol Brain Res* **133:** (2) 299.

Pocernich CB, Boyd-Kimball D, Poon HF, Thongboonkerd V, Lynn BC, Klein JB, Calebrese V, Nath A, Butterfield DA. 2005. Proteomics analysis of human astrocytes expressing the HIV protein Tat. *Mol Brain Res* **133:** (2) 307.

Prasad NB, Biankin AV, Fukushima N, Maitra A, Dhara S, Elkahloun AG, Hruban RH, Goggins N, Leach SD. 2005. Gene expression profiles in pancreatic intraepithelial neoplasia reflect the effects of hedgehog signaling on pancreatic ductal epithelial cells. *Cancer Res* **65:** (5) 1619.

Qian WJ, Jacobs JM, Camp DG, Monroe ME, Moore RJ, Gritsenko MA, Calvano SE, Lowry SF, Xiao WZ, Moldawer LL, Davis RW, Tompkins RG, Smith RD. 2005. Comparative proteome analyses of human plasma following *in vivo* lipopolysaccharide administration using multidimensional separations coupled with tandem mass spectrometry. *Proteomics* **5:** (2) 572.

Quekenborn-Trinquet V, Fogel P, Aldana-Jammayrac O, Ancian P, Demarchez M, Rossio P, Richards HL, Kirby B, Nguyen C, Voegel JJ *et al*. 2005. Gene expression profiles in psoriasis: Analysis of impact of body site location and clinical severity. *Br J Dermatol* **152:** (3) 489.

Rabinowitz YS, Dong LJ, Wistow G. 2005. Gene expression profile studies of human keratoconus cornea for NEIBank: A novel cornea-expressed gene and the absence of transcripts for aquaporin 5. *Invest Ophthalmol Vis Sci* **46:** (4) 1239.

Rakkola R, Matilkainen S, Nyman TA. 2005. Proteome characterization of human MK-92 cells identifies novel IFN-α and IL-15 target genes. *J Proteome Res* **4:** (1) 75.

Ramstrom M, Hagman C, Mitchell JK, Derrick PJ, Hakansson P, Bergquist J. 2005. Depletion of high-abundant proteins in body fluids prior to liquid chromatography Fourier transform ion cyclotron resonance mass spectrometry. *J Proteome Res* **4:** (2) 410.

Ransom RF, Vega-Warner V, Smoyer WE, Klein J. 2005. Differential proteomic analysis of proteins induced by glucocorticoids in cultured murine podocytes. *Kidney Int* **67:** (4) 1275.

Reis EM, Ojopi EPB, Alberto FL, Rahal P, Tsukumo F, Mancini UM, Guimaraes GS, Thompson GMA, Camacho C, Miracca E *et al*. 2005. Large-scale transcriptome analyses reveal new genetic marker candidates of head, neck, and thyroid cancer. *Cancer Res* **65:** (5) 1693.

Relucio KI, Beernink HT, Chen D, Israelski DM, Kim R, Holodniy M. 2005. Proteomic analysis of serum cytokine levels in response to highly active antiretroviral therapy (HAART). *J Proteome Res* **4:** (2) 227.

Rogers JV, Choi YW, Kiser RC, Babin MC, Casillas RP, Schlager JJ, Sabourin CLK. 2004. Microarray analysis of gene expression in murine skin exposed to sulfur mustard. *J Biochem Mol Toxicol* **18:** (6) 289.

Rosengren AT, Nyman TA, Syyrakki S, Matikainen S, Lahesmaa R. 2005. Proteomic and transcriptomic characterization of interferon-α-induced human primary T helper cells. *Proteomics* **5:** (2) 371.

Rossi A, Maione P, Del Gaizo F, Guerriero C, Castaldo V, Gridelli C. 2005. Safety profile of gefitinib in advanced non-small cell lung cancer elderly patients with chronic renal failure: Two clinical cases. *Lung Cancer* **47:** (3) 421.

Rottoli P, Magi B, Perari MG, Liberatori S, Nikiforakis N, Bargagli E, Cianti R, Bini L, Pallini V. 2005. Cytokine profile and proteome analysis in bronchoalveolar lavage of patients with sarcoidosis, pulmonary fibrosis associated with systemic sclerosis and idiopathic pulmonary fibrosis. *Proteomics* **5:** (5) 1423.

Russo D, Bisca A, Celano M, Talamo F, Arturi F, Sciploni A, Presta I, Bulotta S, Ferretti E, Filetti S *et al*. 2005. Proteomic analysis of human thyroid cell lines reveals reduced nuclear localization of Mn-SOD in poorly differentiated thyroid cancer cells. *J Endocrinol Invest* **28:** (2) 137.

Sabourin CLK, Rogers JV, Choi YW, Kiser RC, Casillas RP, Babin MC, Schlager JJ. 2004. Time- and dose-dependent analysis of gene expression using microarrays in sulfur mustard-exposed mice. *J Biochem Mol Toxicol* **18:** (6) 300.

Sauter ER, Shan SM, Hewett JE, Speckman P, Du Bois GC. 2005. Proteomic analysis of nipple aspirate fluid using SELDI-TOF-MS. *Int J Cancer* **114:** (5) 791.

Schaub S, Wilkins JA, Antonovici M, Krokhin O, Weiler T, Rush D, Nickerson P. 2005. Proteomic-based identification of cleaved urinary β2-microglobulin as a potential marker for acute tubular injury in renal allografts. *Am J Transplant* **5:** (4) 729.

Schott P, Singer SS, Kogler H, Neddermeier D, Leineweber K, Brodde OE, Regitz-Zagrosek V, Schmidt B, Dihazi H, Hasenfuss G. 2005.

Copyright © 2006 John Wiley & Sons, Ltd.

Pressure overload and neurohumoral activation differentially affect the myocardial proteome. *Proteomics* **5:** (5) 1372.
Schuetz AN, Yin-Goen Q, Amin MB, Moreno CS, Cohen C, Hornsby CD, Yang WL, Petros JA, Issa MM, Pattaras JG *et al*. 2005. Molecular classification of renal tumors by gene expression profiling. *J Mol Diagn* **7:** (2) 206.
Sgarra R, Tessari MA, Di Bernardo J, Rustighi A, Zago P, Liberatori S, Armini A, Bini L, Giancotti V, Manfioletti G. 2005. Discovering high mobility group A molecular partners in tumour cells. *Proteomics* **5:** (6) 1494.
Shi T, Seligson D, Belldegrun AS, Palotie A, Horvath S. 2005. Tumor classification by tissue microarray profiling: Random forest clustering applied to renal cell carcinoma. *Mod Pathol* **18:** (4) 547.
Sidera C, Parsons R, Austen B. 2005. Post-translational processing of β-secretase in Alzheimer's disease. *Proteomics* **5:** (6) 1533.
Solassol K, Marin P, Demettre E, Rouanet P, Bockaert J, Maudelonde T, Mange A. 2005. Proteomic detection of pro state-specific antigen using a serum fractionation procedure: Potential implication for new low-abundance cancer biomarkers detection. *Anal Biochem* **338:** (1) 26.
Son CG, Bilke S, Davis S, Greer BT, Wei JS, Whiteford CC, Chen QR, Cenacchi N, Khan J. 2005. Database of mRNA gene expression profiles of multiple human organs. *Genome Res* **15:** (3) 443.
Steenman M, Lamirault G, Le Meur N, Le Cunff M, Escande D, Leger JJ. 2005. Distinct molecular portraits of human failing hearts identified by dedicated cDNA microarrays. *Eur J Heart Fail* **7:** (2) 157.
Stekel DJ, Sarti D, Trevino V, Zhang L, Salmon M, Buckley CD, Stevens M, Pallen MJ, Penn C, Falciani F. 2005. Analysis of host response to bacterial infection using error model based gene expression microarray experiments. *Nucleic Acids Res* **33:** (6) e53.
Sukhanov S, Delafontaine P. 2005. Protein chip-based microarray profiling of oxidized low density lipoprotein-treated cells. *Proteomics* **5:** (5) 1274.
Sun JB, Gunzer F, Westendorf AM, Buer J, Scharfe M, Jarek M, Gossling F, Blocker H, Zeng AP. 2005. Genomic peculiarity of coding sequences and metabolic potential of probiotic *Escherichia coli* strain Nissle 1917 inferred from raw genome data. *J Biotechnol* **117:** (2) 147.
Suzuki N, Aoki M, Hinuma Y, Takahashi T, Onodera Y, Ishigaki A, Kato M, Warita H, Tateyama M, Itoyama Y. 2005. Expression profiling with progression of dystrophic change in dysferlin-deficient mice (SJL). *Neurosci Res* **52:** (1) 47.
Szkanderova S, Vavrova J, Hernychova L, Neubauerova V, Lenco J, Stulik J. 2005. Proteome alterations in γ-irradiated human T-lymphocyte leukemia cells. *Radiat Res* **163:** (3) 307.
Tagawa H, Karnan S, Suzuki R, Matsuo K, Zhang XH, Ota A, Morishima Y, Nakamura S, Seto M. 2005. Genome-wide array-based CGH for mantle cell lymphoma: Identification of homozygous deletions of the proapoptotic gene BIM. *Oncogene* **24:** (8) 1348.
Takashima M, Kuramitsu Y, Yokoyama Y, Iizuka N, Fujimoto M, Nishisaka T, Okita K, Oka M, Nakamura K. 2005. Overexpression of α enolase in hepatitis C virus-related hepatocellular carcinoma: Association with tumor progression as determined by proteomic analysis. *Proteomics* **5:** (6) 1686.
Takata R, Katagiri T, Kanehira M, Tsunoda T, Shuin T, Miki T, Namiki M, Kohri K, Matsushita Y, Fujioka T *et al*. 2005. Predicting response to methotrexate, vinblastine, doxorubicin, and cisplatin neoadjuvant chemotherapy for bladder cancers through genome-wide gene expression profiling. *Clin Cancer Res* **11:** (7) 2625.
Talbot SG, Estilo C, Maghami E, Sarkaria IS, Pham DK, O-charoenrat P, Socci ND, Ngai I, Carlson D, Ghossein R *et al*. 2005. Gene expression profiling allows distinction between primary and metastatic squamous cell carcinomas in the lung. *Cancer Res* **65:** (8) 3063.
Tantipaiboonwong P, Sinchaikul S, Sriyam S, Phutrakul S, Chen ST. 2005. Different techniques for urinary protein analysis of normal and lung cancer patients. *Proteomics* **5:** (4) 1140.
Tapio S, Danescu-Mayer J, Asmuss M, Posch A, Gomolka M, Hornhardt S. 2005. Combined effects of γ radiation and arsenite on the proteome of human TK6 lymphoblastoid cells. *Mutat Res* **581:** (1-2) 141.
Teramoto K, Tada M, Tamoto E, Abe M, Kawakami A, Komuro K, Matsunaga A, Shindoh G, Takada M, Murakawa K *et al*. 2005. Prediction of lymphatic invasion/lymph node metastasis, recurrence, and survival in patients with gastric cancer by cDNA array-based expression profiling. *J Surg Res* **124:** (2) 225.
Terashima M, Maesawa C, Oyama K, Ohtani S, Akiyama Y, Ogasawara S, Takagane A, Saito K, Masuda T, Kanzaki N *et al*. 2005. Gene expression profiles in human gastric cancer: Expression of maspin correlates with lymph node metastasis. *Br J Cancer* **92:** (6) 1130.
Tosa M, Ghazizadeh M, Shimizu H, Hirai T, Hyakusoku H, Kawanami O. 2005. Global gene expression analysis of keloid fibroblasts in response to electron beam irradiation reveals the involvement of interleukin-6 pathway. *J Investig Dermatol* **124:** (4) 704.
Tremolada L, Magni F, Valsecchi C, Sarto C, Mocarelli P, Perego R, Cordani N, Favini P, Kienle MG, Sanchez JC *et al*. 2005. Characterization of heat shock protein 27 phosphorylation sites in renal cell carcinoma. *Proteomics* **5:** (3) 788.
Trost M, Wehmhoner D, Karst U, Dieterich G, Wehland J, Jansch L. 2005. Comparative proteome analysis of secretory proteins from pathogenic and nonpathogenic *Listeria* species. *Proteomics* **5:** (6) 1544.
Tsangaris G, Weitzdorfer R, Pollak D, Lubec G, Fountoulakis M. 2005. The amniotic fluid cell proteome. *Electrophoresis* **26:** (6) 1168.
Tyan YC, Wu HY, Su WC, Chen PW, Liao PC. 2005. Proteomic analysis of human pleural effusion. *Proteomics* **5:** (4) 1062.
Unwin RD, Sternberg DW, Lu YN, Pierce A, Gilliland DG, Whetton AD. 2005. Global effects of BCR/ABL and TEL/PDGFRβ expression on the proteome and phosphoproteome - Identification of the rho pathway as a target of BCR/ABL. *J Biol Chem* **280:** (8) 6316.
Urbani A, Poland J, Bernardini S, Bellincampi L, Biroccio A, Schnolzer M, Sinha P, Federici G. 2005. A proteomic investigation into etoposide chemo-resistance of neuroblastoma cell lines. *Proteomics* **5:** (3) 796.
Van't Veer LJ, Paik S, Hayes DF. 2005. Gene expression profiling of breast cancer: A new tumor marker. *J Clin Oncol* **23:** (8) 1631.
Vasudevan SA, Nuchtern JG, Shohet SM. 2005. Gene profiling of high risk neuroblastoma. *World J Surg* **29:** (3) 317.
Wang XP, Li ZJ, Magnusson J, Brunicardi FC. 2005. Tissue MicroArray analyses of pancreatic duodenal homeobox-1 in human cancers. *World J Surg* **29:** (3) 334.
Wang Y, Tetko IV, Hall MA, Frank E, Facius A, Mayer KFX, Mewes HW. 2005. Gene selection from microarray data for cancer classification - A machine learning approach. *Comput Biol Chem* **29:** (1) 37.
Wang ZX, Neuburg D, Li C, Su L, Kim JY, Chen JC, Christiani DC. 2005. Global gene expression profiling in whole-blood samples from individuals exposed to metal fumes. *Environ Health Perspect* **113:** (2) 233.
Weiner CP, Lee KY, Buhimschi CS, Christner R, Buhimschi IA. 2005. Proteomic biomarkers that predict the clinical success of rescue cerclage. *Am J Obstet Gynecol* **192:** (3) 710.
Wellner M, Dechend R, Park JK, Shagdarsuren E, Al-Saadi N, Kirsch T, Gratze P, Schneider W, Meiners S, Fiebeler A *et al*. 2005. Cardiac gene expression profile in rats with terminal heart failure and cachexia. *Physiol Genomics* **20:** (3) 256.
White MY, Cordwell SJ, McCarron HCK, Prasan AM, Craft G, Hambly BD, Jeremy RW. 2005. Proteomics of ischemia/reperfusion injury in rabbit myocardium reveals alterations to proteins of essential functional systems. *Proteomics* **5:** (5) 1395.
Williams DP, Garcia-Allan C, Hanton G, LeNet JL, Provost JP, Brain P, Walsh R, Johnston GI, Smith DA, Park BK. 2004. Time course toxicogenomic profiles in CD-1 mice after nontoxic and nonlethal hepatotoxic paracetamol administration. *Chem Res Toxicol* **17:** (12) 1551.
Wu HFM, Jin M, Marsh CB. 2005. Toward functional proteomics of alveolar macrophages. *Am J Physiol* **288:** (4) L575.
Xia Q, Wang HX, Wang J, Zhang JY, Liu BY, Li AL, Lv M, Hu MR, Yu M, Feng JN *et al*. 2005. Proteomic analysis of interleukin 6-induced differentiation in mouse myeloid leukemia cells. *Int J Biochem Cell Biol* **37:** (6) 1197.
Xu JW, Fan SJ, Rosen EM. 2005. Regulation of the estrogen-inducible gene expression profile by the breast cancer susceptibility gene BRCA1. *Endocrinology* **146:** (4) 2031.
Xu N, Podolsky RH, Chudgar P, Chorich LP, Liu CM, McDonough PG, Warrington JA, Layman LC. 2005. Screening candidate genes for mutations in patients with hypogonadotropic hypogonadism using custom genome resequencing microarrays. *Am J Obstet Gynecol* **192:** (4) 1274.
Yagil C, Hubner N, Monti J, Schulz H, Sapojnikov M, Luft FC, Ganten D, Yagil Y. 2005. Identification of hypertension-related genes through

Copyright © 2006 John Wiley & Sons, Ltd. 

an integrated genomic-transcriptomic approach. *Circ Res* **96:** (6) 617.
Yamada S, Kohu K, Ishii T, Ishidoya S, Hiramatsu M, Kanto S, Fukuzaki A, Adachi Y, Endoh M, Moriya T *et al*. 2005. Gene expression profiling identifies a set of transcripts that are up-regulated in human testicular seminoma. *DNA Res* **11:** (5) 335.
Yamashita S, Suzuki S, Nomoto T, Kondo Y, Wakazono K, Tsujino Y, Sugimura T, Shirai T, Homma Y, Ushijima T. 2005. Linkage and microarray analyses of susceptibility genes in ACI/Seg rats: A model for prostate cancers in the aged. *Cancer Res* **65:** (7) 2610.
Yoshida Y, Miyazaki K, Kamiie J, Sato M, Okuizumi S, Kenmochi A, Kamijo K, Nabetani T, Tsugita A, Xu B *et al*. 2005. Two-dimensional electrophoretic profiling of normal human kidney glomerulus proteome and construction of an extensible markup language (XML)-based database. *Proteomics* **5:** (4) 1083.
Yu JS, Shannon WD, Watson MA, McLeod HL. 2005. Gene expression profiling of the irinotecan pathway in colorectal cancer. *Clin Cancer Res* **11:** (5) 2053.
Zagranichnaya TK, Wu XY, Danos AM, Villereal ML. 2005. Gene expression profiles in HEK-293 cells with low or high store-operated calcium entry: Can regulatory as well as regulated genes be identified? *Physiol Genomics* **21:** (1) 14.
Zenzmaier C, Gesslbauer B, Grobuschek N, Jandrositz A, Preisegger KH, Kungl AJ. 2005. Proteomic profiling of human stem cells derived from umbilical cord blood. *Biochem Biophys Res Commun* **328:** (4) 968.
Zhan XQ, Giorgianni F, Desiderio DM. 2005. Proteomics analysis of growth hormone isoforms in the human pituitary. *Proteomics* **5:** (5) 1228.
Zhang D, Ezekiel UR, Chang SW, Zassenhaus HP. 2005. Gene expression profile in dilated cardiomyopathy caused by elevated frequencies of mitochondrial DNA mutations in the mouse heart. *Cardiovasc Pathol* **14:** (2) 61.

### 8 EST, cDNA and other clone resources

Khabar KSA, Bakheet T, Williams BRG. 2005. AU-rich transient response transcripts in the human genome: Expressed sequence tag clustering and gene discovery approach. *Genomics* **85:** (2) 165.
Nishimura M, Yokoi N, Miki T, Horikawa Y, Yoshioka H, Takeda J, Ohara O, Seino S. 2005. Construction of a multi-functional cDNA library specific for mouse pancreatic islets and its application to microarray. *DNA Res* **11:** (5) 315.
Pavy N, Laroche J, Bousquet J, Mackay J. 2005. Large-scale statistical analysis of secondary xylem ESTs in pine. *Plant Mol Biol* **57:** (2) 203.
Ramirez M, Graham MA, Blanco-Lopez L, Silvente S, Medrano-Soto A, Blair MW, Hernandez G, Vance CP, Lara M. 2005. Sequencing and analysis of common bean ESTs. Building a foundation for functional genomics. *Plant Physiol* **137:** (4) 1211.
Ribichich KF, Salem-Izacc SM, Georg RC, Vencio RZN, Navarro LD, Gomes SL. 2005. Gene discovery and expression profile analysis through sequencing of expressed sequence tags from different developmental stages of the chytridiomycete *Blastocladiella emersonii*. *Eukaryot Cell* **4:** (2) 455.
Siegfried BD, Waterfield N, ffrench-Constant RH. 2005. Expressed sequence tags from *Diabrotica virgifera virgifera* midgut identify a coleopteran cadherin and a diversity of cathepsins. *Insect Mol Biol* **14:** (2) 137.
Sudre K, Leroux C, Cassar-Malek I, Hocquette JF, Martin P. 2005. A collection of bovine cDNA probes for gene expression profiling in muscle. *Mol Cell Probes* **19:** (1) 61.
Zha XF, Xia QY, Zhao P, Li J, Duan J, Wang ZL, Qian JF, Xiang ZH. 2005. Detection and analysis of alternative splicing in the silkworm by aligning expressed sequence tags with the genomic sequence. *Insect Mol Biol* **14:** (2) 113.

### 9 Functional genomics

Brem RB, Kruglyak L. 2005. The landscape of genetic complexity across 5,700 gene expression traits in yeast. *Proc Natl Acad Sci U S A* **102:** (5) 1572.
Miskey C, Izsvak Z, Kawakami K, Ivics Z. 2005. DNA transposons in vertebrate functional genomics. *Cell Mol Life Sci* **62:** (2) 629.
Shearstone JR, Wang YE, Clement A, Allaire NE, Yang CH, Worley DS, Carulli JP, Perrin S. 2005. Application of functional genomic technologies in a mouse model of retinal degeneration. *Genomics* **85:** (3) 309.
Wall DP, Hirsh AE, Fraser HB, Kumm J, Giaever G, Eisen MB, Feldman MW. 2005. Functional genomic analysis of the rates of protein evolution. *Proc Natl Acad Sci U S A* **102:** (15) 5483.

### I0 Transcriptomics

Aguilera J, Petit T, De Winde JH, Pronk JT. 2005. Physiological and genome-wide transcriptional responses of *Saccharomyces cerevisiae* to high carbon dioxide concentrations. *FEMS Yeast Res* **5:** (6-7) 579.
Airik R, Karner M, Karis A, Karner J. 2005. Gene expression analysis of Gata3$^{-/-}$ mice by using cDNA microarray technology. *Life Sci* **76:** (22) 2559.
Andersen MT, Brondsted L, Pearson BM, Mulholland F, Parker M, Pin C, Wells JM, Ingmer H. 2005. Diverse roles for HspR in *Campylobacter jejuni* revealed by the proteome, transcriptome and phenotypic characterization of an hspR mutant. *Microbiology* **151:** (3) 905.
Asamizu E, Nakamura Y, Sato S, Tabata S. 2005. Comparison of the transcript profiles from the root and the nodulating root of the model legume *Lotus japonicus* by serial analysis of gene expression. *Mol Plant Microbe Interact* **18:** (5) 487.
Ashrafuzzaman M, Oh SJ, Hong CB. 2005. Low expression profiles of heat stress-related genes in *Capsicum annuum*. *J Plant Biol* **48:** (1) 85.
Aubin-Horth N, Letcher BH, Hofmann HA. 2005. Interaction of rearing environment and reproductive tactic on gene expression profiles in Atlantic salmon. *J Hered* **96:** (3) 261.
Aziz N, Paiva NL, May GD, Dixon RA. 2005. Transcriptome analysis of alfalfa glandular trichomes. *Planta* **221:** (1) 28.
Bliss TW, Dohms JE, Emara MG, Keeler CL. 2005. Gene expression profiling of avian macrophage activation. *Vet Immunol Immunopathol* **105:** (3-4) 289.
Brandt SP. 2005. Microgenomics: Gene expression analysis at the tissue-specific and single-cell levels. *J Exp Bot* **56:** (412) 495.
Brems S, Guilbride DL, Gundlesdodjir-Planck D, Busold C, Luu VD, Schanne M, Hoheisel J, Clayton C. 2005. The transcriptomes of *Trypanosoma brucei* Lister 427 and TREU927 bloodstream and procyclic trypomastigotes. *Mol Biochem Parasitol* **139:** (2) 163.
Buchanan-Wollaston V, Page T, Harrison E, Breeze E, Lim PO, Nam HG, Lin JF, Wu SH, Swidzinski J, Ishizaki K *et al*. 2005. Comparative transcriptome analysis reveals significant differences in gene expression and signalling pathways between developmental and dark/starvation-induced senescence in *Arabidopsis*. *Plant J* **42:** (4) 567.
Campbell CL, Vandyke KA, Letchworth GJ, Drolet BS, Hanekamp T, Wilson WC. 2005. Midgut and salivary gland transcriptomes of the arbovirus vector *Culicoides sonorensis* (Diptera: Ceratopogonidae). *Insect Mol Biol* **14:** (2) 121.
Daily JP, Le Roch KG, Sarr O, Ndiaye D, Lukens A, Zhou YY, Ndir O, Mboup S, Sultan A, Winzeler EA *et al*. 2005. *In vivo* transcriptome of *Plasmodium falciparum* reveals overexpression of transcripts that encode surface proteins. *J Infect Dis* **191:** (7) 1196.
De Koning DJ, Carlborg O, Haley CS. 2005. The genetic dissection of immune response using gene-expression studies and genome mapping. *Vet Immunol Immunopathol* **105:** (3-4) 343.
Dvorak CMT, Hyland KA, Machado JG, Zhang Y, Fahrenkrug SC, Murtaugh MP. 2005. Gene discovery and expression profiling in porcine Peyer's patch. *Vet Immunol Immunopathol* **105:** (3-4) 301.
El Ouakfaoui S, Miki B. 2005. The stability of the *Arabidopsis* transcriptome in transgenic plants expressing the marker genes nptll and uidA. *Plant J* **41:** (6) 791.
Ernst FD, Bereswill S, Waidner B, Stoof J, Mader U, Kusters JG, Kuipers EJ, Kist M, Van Vliet AHM, Homuth G. 2005. Transcriptional profiling of *Helicobacter pylori* Fur- and iron-regulated gene expression. *Microbiology* **151:** (2) 533.
Eulgem T. 2005. Regulation of the *Arabidopsis* defense transcriptome. *Trends Plant Sci* **10:** (2) 71.
Everts RE, Band MR, Liu ZL, Kumar CG, Liu L, Loor JJ, Oliveira R, Lewin HA. 2005. A 7872-cDNA microarray and its use in bovine functional genomics. *Vet Immunol Immunopathol* **105:** (3-4) 235.
Fitzpatrick JM, Johnston DA, Williams GW, Williams DJ, Freeman TC,

Copyright © 2006 John Wiley & Sons, Ltd.

Dunne DW, Hoffmann KF. 2005. An oligonucleotide microarray for transcriptome analysis of *Schistosoma mansoni* and its application/use to investigate gender-associated gene expression. *Mol Biochem Parasitol* **141:** (1) 1.

Haddadin FT, Harcum SW. 2005. Transcriptome profiles for high-cell-density recombinant and wild-type *Escherichia coli*. *Biotechnol Bioeng* **90:** (2) 127.

Han YP, Zhou DS, Pang X, Zhang L, Song YJ, Tong ZZ, Bao JY, Dai EH, Wang J, Guo ZB *et al*. 2005. Comparative transcriptome analysis of *Yersinia pestis* in response to hyperosmotic and high-salinity stress. *Res Microbiol* **156:** (3) 403.

Hohnjec N, Vieweg ME, Puhler A, Becker A, Kuster H. 2005. Overlaps in the transcriptional profiles of *Medicago truncatula* roots inoculated with two different *Glomus* fungi provide insights into the genetic program activated during arbuscular mycorrhiza (Review). *Plant Physiol* **137:** (4) 1283.

Hyyrylainen HL, Sarvas M, Kontinen VP. 2005. Transcriptome analysis of the secretion stress response of *Bacillus subtilis*. *Appl Microbiol Biotechnol* **67:** (3) 389.

Kovalchuk I, Titov V, Hohn B, Kovalchuka O. 2005. Transcriptome profiling reveals similarities and differences in plant responses to cadmium and lead. *Mutat Res* **570:** (2) 149.

Kurobe T, Yasuike M, Kimura T, Hirono K, Aoki T. 2005. Expression profiling of immune-related genes from Japanese flounder *Paralichthys olivaceus* kidney cells using cDNA microarrays. *Dev Comp Immunol* **29:** (6) 515.

Kusaka M, Yamada K, Kuroyanagi Y, Terauchi A, Kowa H, Kurahashi H, Hoshinaga K. 2005. Gene expression profile in rat renal isografts from brain dead donors. *Transplant Proc* **37:** (1) 364.

Kusakabe Y, Shindo Y, Kim MR, Miura H, Ninomiya Y, Hino A. 2005. cDNA microarray screening for taste-bud-specific genes. *Chem Senses* **30:** (Suppl 1) I12.

Leader DJ. 2005. Transcriptional analysis and functional genomics in wheat. *J Cereal Sci* **41:** (2) 149.

Lee MS, Cho SJ, Tak ES, Lee JA, Cho HJ, Park BJ, Shin C, Kim DK, Park SC. 2005. Transcriptome analysis in the midgut of the earthworm (*Eisenia andrei*) using expressed sequence tags. *Biochem Biophys Res Commun* **328:** (4) 1196.

Liu FL, VanToai T, Moy LP, Bock G, Linford LD, Quackenbush J. 2005. Global transcription profiling reveals comprehensive insights into hypoxic response in *Arabidopsis*. *Plant Physiol* **137:** (3) 1115.

Liu YQ, Gao WM, Wang Y, Wu LY, Liu XD, Yan TF, Alm E, Arkin A, Thompson DK, Fields MW *et al*. 2005. Transcriptome analysis of *Shewanella oneidensis* MR-1 in response to elevated salt conditions. *J Bacteriol* **187:** (7) 2501.

Loreti E, Poggi A, Novi G, Alpi A, Perata P. 2005. A genome-wide analysis of the effects of sucrose on gene expression in *Arabidopsis* seedlings under anoxia. *Plant Physiol* **137:** (3) 1130.

MacFarlane RC, Shah PH, Singh U. 2005. Transcriptional profiling of *Entamoeba histolytica* trophozoites. *Int J Parasitol* **35:** (5) 533.

Marchais V, Kempf M, Licznar P, Lefrancois C, Bouchara JP, Robert R, Cottin J. 2005. DNA array analysis of *Candida albicans* gene expression in response to adherence to polystyrene. *FEMS Microbiol Lett* **245:** (1) 25.

Matsumoto I, Nakamura S, Arai S, Emori Y, Abe K. 2005. DNA microarray analysis of cranial sensory ganglia identifies genes involved in somatosensation in craniofacial structures including oropharynx related to food intake. *Chem Senses* **30:** (Suppl 1)

Mirnics K, Korade Z, Arion D, Lazarov O, Unger T, Macioce M, Sabatini M, Terrano D, Douglass KC, Schor NF *et al*. 2005. Presenilin-1-dependent transcriptome changes. *J Neurosci* **25:** (6) 1571.

Moskvin OV, Gomelsky L, Gomelsky M. 2005. Transcriptome analysis of the *Rhodobacter sphaeroides* PpsR regulon: PpsR as a master regulator of photosystem development. *J Bacteriol* **187:** (6) 2148.

Nakabachi A, Shigenobu S, Sakazume N, Shiraki T, Yayashizaki Y, Carninci P, Ishikawa H, Kudo T, Fukatsu T. 2005. Transcriptome analysis of the aphid bacteriocyte, the symbiotic host cell that harbors an endocellular mutualistic bacterium, *Buchnera*. *Proc Natl Acad Sci U S A* **102:** (15) 5477.

Nishino K, Honda T, Yamaguchi A. 2005. Genome-wide analyses of *Escherichia coli* gene expression responsive to the BaeSR two-component regulatory system. *J Bacteriol* **187:** (5) 1763.

Qiu JF, Zhou DS, Han YP, Zhang L, Tong ZZ, Song YJ, Dai EH, Li B, Wang J, Guo ZB *et al*. 2005. Global gene expression profile of *Yersinia pestis* induced by streptomycin. *FEMS Microbiol Lett* **243:** (2) 489.

Salmon KA, Hung S, Steffen NR, Krupp R, Baldi P, Hatfield GW, Gunsalus RP. 2005. Global gene expression profiling in *Escherichia coli* K12 - Effects of oxygen availability and ArcA. *J Biol Chem* **280:** (15) 15084.

Salunkhe P, Topfer T, Buer J, Tummler B. 2005. Genome-wide transcriptional profiling of the steady-state response of *Pseudomonas aeruginosa* to hydrogen peroxide. *J Bacteriol* **187:** (8) 2565.

Schlingemann J, Thuerigen O, Ittrich C, Toedt G, Kramer H, Hahn M, Lichter P. 2005. Effective transcriptome amplification for expression profiling on sense-oriented oligonucleotide microarrays. *Nucleic Acids Res* **33:** (3) e29.

Smajs D, McKevitt M, Howell JK, Norris SJ, Cai WW, Palzkill T, Weinstock GM. 2005. Transcriptome of *Treponema pallidum*: Gene expression profile during experimental rabbit infection. *J Bacteriol* **187:** (5) 1866.

Steil L, Serrano M, Henriques AO, Volker U. 2005. Genome-wide analysis of temporally regulated and compartment-specific gene expression in sporulating cells of *Bacillus subtilis*. *Microbiology* **151:** (2) 399.

Stintzi A, Marlow D, Palyada K, Naikare H, Panciera R, Whitworth L, Clarke C. 2005. Use of genome-wide expression profiling and mutagenesis to study the intestinal lifestyle of *Campylobacter jejuni*. *Infect Immun* **73:** (3) 1797.

Stolc V, Samanta MP, Tongprasit W, Marshall WF. 2005. Genome-wide transcriptional analysis of flagellar regeneration in *Chlamydomonas reinhardtii* identifies orthologs of ciliary disease genes. *Proc Natl Acad Sci U S A* **102:** (10) 3703.

Stolc V, Samanta MP, Tongprasit W, Sethi H, Liang SD, Nelson DC, Hegeman A, Nelson C, Rancour D, Bednarek S *et al*. 2005. Identification of transcribed sequences in *Arabidopsis thaliana* by using high-resolution genome tiling arrays. *Proc Natl Acad Sci U S A* **102:** (12) 4453.

Suleau A, Jacques N, Reitz-Ausseur J, Casaregola S. 2005. Intraspecific gene expression variability in the yeast *Kluyveromyces lactis* revealed by micro-array analysis. *FEMS Yeast Res* **5:** (6-7) 595.

Tachibana C, Yoo JY, Tagne JB, Kacherovsky N, Lee TI, Young ET. 2005. Combined global localization analysis and transcriptome data identify genes that are directly coregulated by Adr1 and Cat8. *Mol Cell Biol* **25:** (6) 2138.

Thomas N, Kenrick M, Giesler T, Kiser G, Tinkler H, Stubbs S. 2005. Characterization and gene expression profiling of a stable cell line expressing a cell cycle GFP sensor. *Cell Cycle* **4:** (1) 191.

Tran LM, Brynildsen MP, Kao KC, Suen JK, Liao JC. 2005. gNCA: A framework for determining transcription factor activity based on transcriptome: Identifiability and numerical implementation. *Metab Eng* **7:** (2) 128.

Viguerie N, Poitou C, Cancello R, Stich V, Clement K, Langin D. 2005. Transcriptomics applied to obesity and caloric restriction. *Biochimie* **87:** (1) 117.

Wang YH, Byrne KA, Reverter A, Harper GS, Taniguchi M, McWilliam SM, Mannen H, Oyama K, Lehnert SA. 2005. Transcriptional profiling of skeletal muscle tissue from two breeds of cattle. *Mamm Genome* **16:** (3) 201.

Weber H, Polen T, Heuveling J, Wendisch VF, Hengge R. 2005. Genome-wide analysis of the general stress response network in *Escherichia coli*: $\sigma^S$-dependent genes, promoters, and sigma factor selectivity. *J Bacteriol* **187:** (5) 1591.

Wittenberg C, Reed SI. 2005. Cell cycle-dependent transcription in yeast: Promoters, transcription factors, and transcriptomes. *Oncogene* **24:** (17) 2746.

Yang KA, Lim CJ, Hong JK, Jin ZL, Hong JC, Yun DJ, Chung WS, Lee SY, Cho MJ, Lim CO. 2005. Identification of Chinese cabbage genes up-regulated by prolonged cold by using microarray analysis. *Plant Sci* **168:** (4) 959.

Zhou Q, Shima JE, Nie R, Friel PJ, Griswold MD. 2005. Androgen-regulated transcripts in the neonatal mouse testis as determined through microarray analysis. *Biol Reprod* **72:** (4) 1010.

Zierold U, Scholz U, Schweizer P. 2005. Transcriptome analysis of *mlo*-mediated resistance in the epidermis of barley. *Mol Plant Pathol* **6:** (2) 139.

Copyright © 2006 John Wiley & Sons, Ltd. 

### II Proteomics

Aldor IS, Krawitz DC, Forrest W, Chen C, Nishihara JC, Joly JC, Champion KM. 2005. Proteomic profiling of recombinant *Escherichia coli* in high-cell-density fermentations for improved production of an antibody fragment biopharmaceutical. *Appl Environ Microbiol* **71:** (4) 1717.

Arevalo-Ferro C, Reil G, Gorg A, Eberl L, Riedel K. 2005. Biofilm formation of *Pseudomonas putida* IsoF: The role of quorum sensing as assessed by proteomics. *Syst Appl Microbiol* **28:** (2) 87.

Babic N, Beeson C, Dovichi NJ. 2005. Effect of high density lipoproteins on protein expression in myoblast cell lines. *J Proteome Res* **4:** (2) 344.

Backert S, Kwok T, Schmid M, Selbach M, Moese S, Peek RM, Konig W, Meyer TF, Jungblut PR. 2005. Subproteomes of soluble and structure-bound *Helicobacter pylori* proteins analyzed by two-dimensional gel electrophoresis and mass spectrometry. *Proteomics* **5:** (5) 1331.

Balyasnikova IV, Woodman ZL, Albrecht RF, Natesh R, Acharya KR, Sturrock ED, Danilov SM. 2005. Localization of an N-domain region of angiotensin-converting enzyme involved in the regulation of ectodomain shedding using monoclonal antibodies. *J Proteome Res* **4:** (2) 258.

Baussant T, Bougueleret L, Johnson A, Rogers J, Menin L, Hall M, Aberg PM, Rose K. 2005. Effective depletion of albumin using a new peptide-based affinity medium. *Proteomics* **5:** (4) 973.

Beckner ME, Chen X, An JY, Day BW, Pollack IF. 2005. Proteomic characterization of harvested pseudopodia with differential gel electrophoresis and specific antibodies. *Lab Invest* **85:** (3) 316.

Biron DG, Joly C, Galeotti N, Ponton F, Marche L. 2005. The proteomics: A new prospect for studying parasitic manipulation. *Behav Process* **68:** (3) 249.

Blonder J, Hale ML, Chan KC, Yu LR, Lucas DA, Conrads TP, Zhou M, Popoff MR, Issaq HJ, Stiles BG *et al*. 2005. Quantitative profiling of the detergent-resistant membrane proteome of ι-b toxin induced Vero cells. *J Proteome Res* **4:** (2) 523.

Boldt A, Fortunato D, Conti A, Petersen A, Ballmer-Weber B, Lepp U, Reese G, Becker WM. 2005. Analysis of the composition of an immunoglobulin E reactive high molecular weight protein complex of peanut extract containing Ara h 1 and Ara h 3/4. *Proteomics* **5:** (3) 675.

Booy AT, Haddow JD, Ohlund LB, Hardie DB, Olafson RW. 2005. Application of isotope coded affinity tag (ICAT) analysis for the identification of differentially expressed proteins following infection of Atlantic salmon (*Salmo salar*) with infectious hematopoietic necrosis virus (IHNV) or *Renibacterium salmoninarum* (BKD). *J Proteome Res* **4:** (2) 325.

Bosworth CA, Chou CW, Cole RB, Rees BB. 2005. Protein expression patterns in zebrafish skeletal muscle: Initial characterization and the effects of hypoxic exposure. *Proteomics* **5:** (5) 1362.

Bouley J, Meunier B, Chambon C, De Smet S, Hocquette JF, Picard B. 2005. Proteomic analysis of bovine skeletal muscle hypertrophy. *Proteomics* **5:** (2) 490.

Boyd-Kimball D, Sultana R, Poon HF, Mohmmad-Abdul H, Lynn BC, Klein JB, Butterfield DA. 2005. γ-Glutamylcysteine ethyl ester protection of proteins from Aβ(1-42)-mediated oxidative stress in neuronal cell culture: A proteomics approach. *J Neurosci Res* **79:** (5) 707.

Caffarri S, Frigerio S, Olivieri E, Righetti PG, Bassi R. 2005. Differential accumulation of *Lhcb* gene products in thylakoid membranes of *Zea mays* plants grown under contrasting light and temperature conditions. *Proteomics* **5:** (3) 758.

Cai YD, Chou KC. 2005. Using functional domain composition to predict enzyme family classes. *J Proteome Res* **4:** (1) 109.

Calvo E, Pucciarelli MG, Bierne H, Cossart P, Albar JP, Garcia-del Portilla F. 2005. Analysis of the *Listeria* cell wall proteome by two-dimensional nanoliquid chromatography coupled to mass spectrometry. *Proteomics* **5:** (2) 433.

Chan LL, Hodgkiss IJ, Lam PKS, Wan JMF, Chou HN, Lum JHK, Lo MGY, Mak ASC, Sit WH, Lo SCL. 2005. Use of two-dimensional gel electrophoresis to differentiate morphospecies of *Alexandrium minutum*, a paralytic shellfish poisoning toxin-producing dinoflagellate of harmful algal blooms. *Proteomics* **5:** (6) 1580.

Chang IF, Szick-Miranda K, Pan SQ, Bailey-Serres J. 2005. Proteomic characterization of evolutionarily conserved and variable proteins of *Arabidopsis* cytosolic ribosomes. *Plant Physiol* **137:** (3) 848.

Chen XL, Cushman SW, Pannell LK, Hess S. 2005. Quantitative proteomic analysis of the secretory proteins from rat adipose cells using a 2D liquid chromatography-MS/MS approach. *J Proteome Res* **4:** (2) 570.

Cheng GF, Lin JJ, Feng XG, Fu ZQ, Jin YM, Yuan CX, Zhou YC, Cai YM. 2005. Proteomic analysis of differentially expressed proteins between the male and female worm of *Schistosomal japonicum* after pairing. *Proteomics* **5:** (2) 511.

Choi JY, Joo WA, Park SJ, Lee SH, Kim CW. 2005. An efficient proteomics based strategy for the functional characterization of a novel halophilic enzyme from *Halobacterium salinarum*. *Proteomics* **5:** (4) 907.

Ciambella C, Roepstorff P, Aro EM, Zolla L. 2005. A proteomic approach for investigation of photosynthetic apparatus in plants. *Proteomics* **5:** (3) 746.

Colinge J, Cusin I, Reffas S, Mahe E, Niknejad A, Rey PA, Mattou H, Moniatte M, Bougueleret L. 2005. Experiments in searching small proteins in unannotated large eukaryotic genomes. *J Proteome Res* **4:** (1) 167.

Collins MO, Yu L, Coba MP, Husi H, Campuzano L, Blackstock WP, Choudhary JS, Grant SGN. 2005. Proteomic analysis of *in vivo* phosphorylated synaptic proteins. *J Biol Chem* **280:** (7) 5972.

Corti V, Cattaneo A, Bachi A, Rossi RE, Monasterolo G, Paolucci C, Burastero SE, Alessio M. 2005. Identification of grass pollen allergens by two-dimensional gel electrophoresis and serological screening. *Proteomics* **5:** (3) 729.

Dani V, Simon WJ, Duranti M, Croy RRD. 2005. Changes in the tobacco leaf apoplast proteome in response to salt stress. *Proteomics* **5:** (3) 737.

De Vriendt K, Theunissen S, Carpentier W, De Smet L, Devreese B, Van Beeumen J. 2005. Proteomics of *Shewanella oneidensis* MR-1 biofilm reveals differentially expressed proteins, including AggA and RibB. *Proteomics* **5:** (5) 1308.

Donnelly BE, Madden RD, Ayoubi P, Porter DR, Dillwith JW. 2005. The wheat (*Triticum aestivium* L) leaf proteome. *Proteomics* **5:** (6) 1624.

Dreger M, Mika J, Bieller A, Jahnel R, Gillen C, Schaefer MKH, Weihe E, Hucho F. 2005. Analysis of the dorsal spinal cord synaptic architecture by combined proteome analysis and *in situ* hybridization. *J Proteome Res* **4:** (2) 238.

Flaete NES, Hollung K, Ruud L, Sogn T, Faergestad EM, Skarpeid HJ, Magnus EM, Uhlen AK. 2005. Combined nitrogen and sulphur fertilisation and its effect on wheat quality and protein composition measured by SE-FPLC and proteomics. *J Cereal Sci* **41:** (3) 357.

Fountoulakis M, Soumaka E, Rapti K, Mavroidis M, Tsangaris G, Maris A, Weisleder N, Capetanaki Y. 2005. Alterations in the heart mitochondrial proteome in a desmin null heart failure model. *J Mol Cell Cardiol* **38:** (3) 461.

Fountoulakis M, Tsangaris G, Maris A, Lubec G. 2005. The rat brain hippocampus proteome. *J Chromatogr B* **819:** (1) 115.

Francis AW, Ruggiero CE, Koppisch AT, Dong JG, Song J, Brettin T, Iyer S. 2005. Proteomic analysis of *Bacillus anthracis* Sterne vegetative cells. *Biochim Biophys Acta* **1748:** (2) 191.

Fry BG. 2005. From genome to "venome": Molecular origin and evolution of the snake venom proteome inferred from phylogenetic analysis of toxin sequences and related body proteins. *Genome Res* **15:** (3) 403.

Gendrel AV, Lippman Z, Martienssen R, Colot V. 2005. Profiling histone modification patterns in plants using genomic tiling microarrays. *Nat Methods* **2:** (3) 213.

Gil-Agusti MT, Campostrini N, Zolla L, Ciambella C, Invernizzi C, Righetti PG. 2005. Two-dimensional mapping as a tool for classification of green coffee bean species. *Proteomics* **5:** (3) 710.

Goodchild A, Raftery M, Saunders NFW, Guilhaus M, Cavicchioli R. 2005. Cold adaptation of the Antarctic archaeon, *Methanococcoides burtonii* assessed by proteomics using ICAT. *J Proteome Res* **4:** (2) 473.

Gosalia DN, Salisbury CM, Maly DJ, Ellman JA, Diamond SL. 2005. Profiling serine protease substrate specificity with solution phase fluorogenic peptide microarrays. *Proteomics* **5:** (5) 1292.

Grant MM, Barber VS, Griffiths HR. 2005. The presence of ascorbate induces expression of brain derived neurotrophic factor in SH-SY5Y neuroblastoma cells after peroxide insult, which is associated with increased survival. *Proteomics* **5:** (2) 534.

Copyright © 2006 John Wiley & Sons, Ltd.

Gutjahr C, Murphy D, Lueking A, Koenig A, Janitz M, O'Brien J, Korn B, Horn S, Lehrach H, Cahill DJ. 2005. Mouse protein arrays from a $T_H1$ cell cDNA library for antibody screening and serum profiling. *Genomics* **85:** (3) 285.
Hajduch M, Ganapathy A, Stein JW, Thelen JJ. 2005. A systematic proteomic study of seed filling in soybean. Establishment of high-resolution two-dimensional reference maps, expression profiles, and an interactive proteome database. *Plant Physiol* **137:** (4) 1397.
Hajheidari M, Abdollahian-Noghabi M, Askari H, Heidari M, Sadeghian SY, Ober ES, Salekdeh GH. 2005. Proteome analysis of sugar beet leaves under drought stress. *Proteomics* **5:** (4) 950.
Hancock WS. 2005. Toward a common proteomics platform. *J Proteome Res* **4:** (2) 213.
Holland JW, Deeth HC, Alewood PF. 2005. Analysis of *O*-glycosylation site occupancy in bovine κ-casein glycoforms separated by two-dimensional gel electrophoresis. *Proteomics* **5:** (4) 990.
Karadzic IM, Maupin-Furlow JA. 2005. Improvement of two-dimensional gel electrophoresis proteome maps of the haloarchaeon *Haloferax volcanii*. *Proteomics* **5:** (2) 354.
Kim SH, Jun S, Jang HS, Lim SK. 2005. Identification of parathyroid hormone-regulated proteins in mouse bone marrow cells by proteomics. *Biochem Biophys Res Commun* **330:** (2) 423.
Kuznetsova IM, Stepanenko OV, Turoverov KK, Staiano M, Scognamiglio V, Rossi M, D'Auria S. 2005. Fluorescence properties of glutamine-binding protein from *Escherichia coli* and its complex with glutamine. *J Proteome Res* **4:** (2) 417.
Lewenza S, Gardy JL, Brinkman FSL, Hancock REW. 2005. Genome-wide identification of *Pseudomonas aeruginosa* exported proteins using a consensus computational strategy combined with a laboratory-based PhoA fusion screen. *Genome Res* **15:** (2) 321.
Li QB, Li LY, Rejtar T, Karger BL, Ferry JG. 2005. Proteome of *Methanosarcina acetivorans* Part I: An expanded view of the biology of the cell. *J Proteome Res* **4:** (1) 112.
Li QB, Li LY, Rejtar T, Karger BL, Ferry JG. 2005. Proteome of *Methanosarcina acetivorans* Part II: Comparison of protein levels in acetate- and methanol-grown cells. *J Proteome Res* **4:** (1) 129.
Lindermayr C, Saalbach G, Durner J. 2005. Proteomic identification of S-nitrosylated proteins in *Arabidopsis*. *Plant Physiol* **137:** (3) 921.
Liu S, Zhang C, Zhou YQ. 2005. Domain graph of *Arabidopsis* proteome by comparative analysis. *J Proteome Res* **4:** (2) 435.
Luppens SBI, Ten Cate JM. 2005. Effect of biofilm model, mode of growth, and strain on *Streptococcus mutans* protein expression as determined by two dimensional difference gel electrophoresis. *J Proteome Res* **4:** (2) 232.
Maeda K, Finnie C, Svensson B. 2005. Identification of thioredoxin h-reducible disulphides in proteomes by differential labelling of cysteines: Insight into recognition and regulation of proteins in barley seeds by thioredoxin h. *Proteomics* **5:** (6) 1634.
McCormick DJ, Holmes MW, Muddiman DC, Madden BJ. 2005. Mapping sites of protein phosphorylation by mass spectrometry utilizing a chemical enzymatic approach: Characterization of products from α-S1casein phosphopeptides. *J Proteome Res* **4:** (2) 424.
Muccilli V, Cunsolo V, Saletti R, Foti S, Masci S, Lafiandra D. 2005. Characterization of B- and C-type low molecular weight glutenin subunits by electrospray ionization mass spectrometry and matrix-assisted laser desorption/ionization mass spectrometry. *Proteomics* **5:** (3) 719.
Nandakumar R, Nandakumar MP, Marten MR, Ross JM. 2005. Proteome analysis of membrane and cell wall associated proteins from *Staphylococcus aureus*. *J Proteome Res* **4:** (2) 250.
Neo JCH, Rose P, Ong CN, Chung MCM. 2005. β-Phenylethyl isothiocyanate mediated apoptosis: A proteomic investigation of early apoptotic protein changes. *Proteomics* **5:** (4) 1075.
Ocana MF, Jarvis J, Parker R, Bramley PM, Halket JM, Patel RKP, Neubert H. 2005. C-terminal sequencing by mass spectrometry: Application to gelatine-derived proline-rich peptides. *Proteomics* **5:** (5) 1209.
Okada M, Huston CD, Mann BJ, Petri WA, Kita K, Nozaki T. 2005. Proteomic analysis of phagocytosis in the enteric protozoan parasite *Entamoeba histolytica*. *Eukaryot Cell* **4:** (4) 827.
Okerberg ES, Wu JY, Zhang BH, Samii B, Blackford K, Winn DT, Shreder KR, Burbaum JJ, Patricelli MP. 2005. High-resolution functional proteomics by active-site peptide profiling. *Proc Natl Acad Sci U S A* **102:** (14) 4996.
Pessione E, Mazzoli R, Giuffrida MG, Lamberti C, Garcia-Moruno E, Barello C, Conti A, Giunta C. 2005. A proteomic approach to studying biogenic amine producing lactic acid bacteria. *Proteomics* **5:** (3) 687.
Phinney BS, Thelen JJ. 2005. Proteomic characterization of a Triton-insoluble fraction from chloroplasts defines a novel group of proteins associated with macromolecular structures. *J Proteome Res* **4:** (2) 497.
Pierson J, Svenningsson P, Caprioli RM, Andren PE. 2005. Increased levels of ubiquitin in the 6-OHDA-lesioned striatum of rats. *J Proteome Res* **4:** (2) 223.
Ponamarev MV, She YM, Zhang L, Robinson BH. 2005. Proteomics of bovine mitochondrial RNA-binding proteins: HES1/KNP-I is a new mitochondrial resident protein. *J Proteome Res* **4:** (1) 43.
Rahman MA, Isa Y, Uehara T. 2005. Proteins of calcified endoskeleton: II Partial amino acid sequences of endoskeletal proteins and the characterization of proteinaceous organic matrix of spicules from the alcyonarian, *Synularia polydactyla*. *Proteomics* **5:** (4) 885.
Rao KCS, Carruth RT, Miyagi M. 2005. Proteolytic $^{18}O$ labeling by peptidyl-Lys metalloendopeptidase for comparative proteomics. *J Proteome Res* **4:** (2) 507.
Ravichandran V, Sriram RD. 2005. Toward data standards for proteomics. *Nat Biotechnol* **23:** (3) 373.
Renesto P, Azza S, Dolla A, Fourquet P, Vestris G, Gorvel JP, Raoult D. 2005. Proteome analysis of *Rickettsia conorii* by two-dimensional gel electrophoresis coupled with mass spectrometry. *FEMS Microbiol Lett* **245:** (2) 231.
Salek M, Lehmann WD. 2005. Analysis of thyroglobulin iodination by tandem mass spectrometry using immonium ions of monoiodo- and diiodo-tyrosine. *Proteomics* **5:** (2) 351.
Salt LJ, Robertson JA, Jenkins JA, Mulholland F, Mills ENC. 2005. The identification of foam-forming soluble proteins from wheat (*Triticum aestivum*) dough. *Proteomics* **5:** (6) 1612.
Samyn B, Sargeant K, Castanheira P, Faro C, Van Beeumen J. 2005. A new method for C-terminal sequence analysis in the proteomic era. *Nat Methods* **2:** (3) 193.
Sanchez S, Arenas J, Abel A, Criado MT, Ferreiros CM. 2005. Analysis of outer membrane protein complexes and heat-modifiable proteins in *Neisseria* strains using two-dimensional diagonal electrophoresis. *J Proteome Res* **4:** (1) 91.
Satoh M, Haruta-Satoh E, Omori A, Oh-Ishi M, Kodera Y, Furudate SI, Maeda T. 2005. Effect of thyroxine on abnormal pancreatic proteomes of the hypothyroid *rdw* rat. *Proteomics* **5:** (4) 1113.
Saunders NFW, Goodchild A, Raftery M, Guilhaus M, Curmi PMG, Cavicchioli R. 2005. Predicted roles for hypothetical proteins in the low-temperature expressed proteome of the Antarctic archaeon *Methanococcoides burtonii*. *J Proteome Res* **4:** (2) 464.
Scarselli R, Donadio E, Giuffrida MG, Fortunato D, Conti A, Balestreri E, Felicioli R, Pinzauti M, Sabatini AG, Felicioli A. 2005. Towards royal jelly proteome. *Proteomics* **5:** (3) 769.
Schaefer H, Chamrad DC, Marcus K, Reidegeld KA, Bluggel M, Meyer HE. 2005. Tryptic transpeptidation products observed in proteome analysis by liquid chromatography-tandem mass spectrometry. *Proteomics* **5:** (4) 846.
Schluesener D, Fischer F, Kruip J, Rogner M, Poetsch A. 2005. Mapping the membrane proteome of *Corynebacterium glutamicum*. *Proteomics* **5:** (5) 1317.
Serrano SMT, Shannon JD, Wang DY, Camargo ACM, Fox JW. 2005. A multifaceted analysis of viperid snake venoms by two-dimensional gel electrophoresis: An approach to understanding venom proteomics. *Proteomics* **5:** (2) 501.
Shen XC, Valencia CA, Szostak J, Dong B, Liu RH. 2005. Scanning the human proteome for calmodulin-binding proteins. *Proc Natl Acad Sci U S A* **102:** (17) 5969.
Skipp P, Robinson J, O'Connor CD, Clarke IN. 2005. Shotgun proteomic analysis of *Chlamydia trachomatis*. *Proteomics* **5:** (6) 1558.
Skylas D, Van Dyk D, Wrigley CW. 2005. Proteomics of wheat grain. *J Cereal Sci* **41:** (2) 165.
Skylas DJ, Cordwell SJ, Craft G, McInerney B, Wu MJ, Chin J, Wrigley CW, Sharp PJ. 2005. Proteome analysis of two soft-grained wheats of different processing quality: Cultivar-specific proteins. *Aust J Agric Res* **56:** (2) 145.
Sleat DE, Lackland H, Wang YH, Sohar I, Xiao G, Li H, Lobel P. 2005. The human brain mannose 6-phosphate glycoproteome: A complex mixture composed of multiple isoforms of many soluble lysosomal

Copyright © 2006 John Wiley & Sons, Ltd. 

proteins. *Proteomics* **5:** (6) 1520.
Stupak J, Liu HZ, Wang ZP, Brix BJ, Fliegel L, Li L. 2005. Nanoliter sample handling combined with microspot MALDI-MS for detection of gel-separated phosphoproteins. *J Proteome Res* **4:** (2) 515.
Sun NK, Jang J, Lee S, Kim S, Lee S, Hoe KL, Chung KS, Kim DU, Yoo HS, Won M *et al*. 2005. The first two-dimensional reference map of the fission yeast, *Schizosaccharomyces pombe* proteins. *Proteomics* **5:** (6) 1574.
Tackett AJ, Dilworth DJ, Davey MJ, O'Donnell M, Aitchison JD, Rout MP, Chait BT. 2005. Proteomic and genomic characterization of chromatin complexes at a boundary. *J Cell Biol* **169:** (1) 35.
Tang TK, Wu MPJ, Chen ST, Hou MH, Hong MH, Pan FM, Yu HM, Chen JH, Yao CW, Wang AHJ. 2005. Biochemical and immunological studies of nucleocapsid proteins of severe acute respiratory syndrome and 229E human coronaviruses. *Proteomics* **5:** (4) 925.
Todd JD, Sawers G, Johnston AWB. 2005. Proteomic analysis reveals the wide-ranging effects of the novel, iron-responsive regulator RirA in *Rhizobium leguminosarum* bv. *viciae*. *Mol Genet Genomics* **273:** (2) 197.
Vandenbrouck Y, Garin J, Jaquinod M, Bruley C. 2005. Proteomics: Data analysis of mass spectrometry results (French). *Biofutur* **252:** 27.
Vazquez-Pianzola P, Urlaub H, Rivera-Pomar R. 2005. Proteomic analysis of reaper 5′ untranslated region-interacting factors isolated by tobramycin affinity-selection reveals a role for La antigen in *reaper* mRNA translation. *Proteomics* **5:** (6) 1645.
Verhelst SHL, Bogyo M. 2005. Dissecting protein function using chemical proteomic methods. *QSAR Comb Sci* **24:** (2) 261.
Vosseller K, Hansen KC, Chalkley RJ, Trinidad JC, Wells L, Hart GW, Burlingame AL. 2005. Quantitative analysis of both protein expression and serine/threonine post-translational modifications through stable isotope labeling with dithiothreitol. *Proteomics* **5:** (2) 388.
Wan JR, Torres M, Ganapathy A, Thelen J, DaGue BB, Mooney B, Xu D, Stacey G. 2005. Proteomic analysis of soybean root hairs after infection by *Bradyrhizobium japonicum*. *Mol Plant Microbe Interact* **18:** (5) 458.
Wang HYJ, Taggi AE, Meinwald J, Wise RA, Woods AS. 2005. Study of the interaction of chlorisondamine and chlorisondamine analogues with an epitope of the α-2 neuronal acetylcholine nicotinic receptor subunit. *J Proteome Res* **4:** (2) 532.
Wang YJ, Balgley BM, Rudnick PA, Evans EL, DeVoe DL, Lee CS. 2005. Integrated capillary isoelectric focusing/nano-reversed phase liquid chromatography coupled with ESI-MS for characterization of intact yeast proteins. *J Proteome Res* **4:** (1) 36.
Weeks ME, Sinclair J, Jacob RJ, Saxton MJ, Kirby S, Jones J, Waterfield MD, Cramer R, Timms JF. 2005. Stress-induced changes in the *Schizosaccharomyces pombe* proteome using two-dimensional difference gel electrophoresis, mass spectrometry and a novel integrated robotics platform. *Proteomics* **5:** (6) 1669.
Wuchty S, Almaas E. 2005. Peeling the yeast protein network. *Proteomics* **5:** (2) 444.
Yang GX, Inoue A, Takasaki H, Kaku H, Akao S, Komatsu S. 2005. Proteomic approach to analyze auxin- and zinc-responsive protein in rice. *J Proteome Res* **4:** (2) 456.
Yi EC, Li XJ, Cooke K, Lee H, Raught B, Page A, Aneliunas V, Hieter P, Goodlett DR, Aebersold R. 2005. Increased quantitative proteome coverage with $^{13}C/^{12}C$-based, acid-cleavable isotope-coded affinity tag reagent and modified data acquisition scheme. *Proteomics* **5:** (2) 380.
Zhang B, Su YP, Wang FC, Ai GP, Wei YJ. 2005. Identification of differentially expressed proteins of γ-ray irradiated rat intestinal epithelial IEC-6 cells by two-dimensional gel electrophoresis and matrix-assisted laser desorption/ionisation-time of flight mass spectrometry. *Proteomics* **5:** (2) 426.

### I2 Protein structural genomics

Oldfield CJ, Ulrich EL, Cheng YG, Dunker AK, Markley JL. 2005. Addressing the intrinsic disorder bottleneck in structural proteomics. *Proteins* **59:** (3) 444.
Page R, Peti W, Wilson IA, Stevens RC, Wuthrich K. 2005. NMR screening and crystal quality of bacterially expressed prokaryotic and eukaryotic proteins in a structural genomics pipeline. *Proc Natl Acad Sci U S A* **102:** (6) 1901.

### I3 Metabolomics

Bollard ME, Keun HC, Beckonert O, Ebbels TMD, Antti H, Nicholls AW, Shockcor JP, Cantor GH, Stevens G, Lindon JC *et al*. 2005. Comparative metabonomics of differential hydrazine toxicity in the rat and mouse. *Toxicol Appl Pharmacol* **204:** (2) 135.
Patil KR, Nielsen J. 2005. Uncovering transcriptional regulation of metabolism by using metabolic network topology. *Proc Natl Acad Sci U S A* **102:** (8) 2685.
Tohge T, Nishiyama Y, Hirai MY, Yano M, Nakajima J, Awazuhara M, Inoue E, Takahashi H, Goodenowe DB, Kitayama M *et al*. 2005. Functional genomics by integrated analysis of metabolome and transcriptome of *Arabidopsis* plants over-expressing an MYB transcription factor. *Plant J* **42:** (2) 218.

### I4 Genomic approaches to development

Alonso J, Santaren JF. 2005. Proteomic analysis of the wing imaginal discs of *Drosophila melanogaster*. *Proteomics* **5:** (2) 474.
An J, Yuan Q, Wang C, Li L, Ke T, Tian HY, Jing NH, Zhao FK. 2005. Differential display of proteins involved in the neural differentiation of mouse embryonic carcinoma P19 cells by comparative proteomic analysis. *Proteomics* **5:** (6) 1656.
Baker MA, Witherdin R, Hetherington L, Cunningham-Smith K, Aitken RJ. 2005. Identification of post-translational modifications that occur during sperm maturation using difference in two-dimensional gel electrophoresis. *Proteomics* **5:** (4) 1003.
Baldessari D, Shin Y, Krebs O, Konig R, Koide T, Vinayagam A, Fenger U, Mochii M, Terasaka C, Kitayama A *et al*. 2005. Global gene expression profiling and cluster analysis in *Xenopus laevis*. *Mech Dev* **122:** (3) 441.
Bystrykh L, Weersing E, Dontje B, Sutton S, Pletcher MT, Wiltshire T, Su AI, Vellenga E, Wang JT, Manly KF *et al*. 2005. Uncovering regulatory pathways that affect hematopoietic stem cell function using 'genetical genomics'. *Nat Genet* **37:** (3) 225.
Chalmers AD, Goldstone K, Smith JC, Gilchrist M, Amaya E, Papalopulu N. 2005. A *Xenopus tropicalis* oligonucleotide microarray works across species using RNA from *Xenopus laevis*. *Mech Dev* **122:** (3) 355.
Chen JA, Voigt J, Gilchrist M, Papalopulu N, Amaya E. 2005. Identification of novel genes affecting mesoderm formation and morphogenesis through an enhanced large scale functional screen in *Xenopus*. *Mech Dev* **122:** (3) 307.
DasGupta R, Kaykas A, Moon RT, Perrimon N. 2005. Functional genomic analysis of the Wnt-Wingless signaling pathway. *Science* **308:** (5723) 826.
De Groot CJM, Steegers-Theunissen RP, Guzel CK, Steegers EAP, Luider TM. 2005. Peptide patterns of laser dissected human trophoblasts analyzed by matrix-assisted laser desorption/ionisation-time of flight mass spectrometry. *Proteomics* **5:** (2) 597.
Donaldson IJ, Chapman M, Kinston S, Landry JR, Knezevic K, Piltz S, Buckley N, Green AR, Gottgens B. 2005. Genome-wide identification of *cis*-regulatory sequences controlling blood and endothelial development. *Hum Mol Genet* **14:** (5) 595.
Getchell TV, Liu H, Vaishnav RA, Kwong K, Stromberg AJ, Getchell ML. 2005. Temporal profiling of gene expression during neurogenesis and remodeling in the olfactory epithelium at short intervals after target ablation. *J Neurosci Res* **80:** (3) 309.
Goodisman MAD, Isoe J, Wheeler DE, Wells MA. 2005. Evolution of insect metamorphosis: A microarray-based study of larval and adult gene expression in the ant *Camponotus festinatus*. *Evolution* **59:** (4) 858.
Herrera L, Ottolenghi C, Garcia-Ortiz JE, Pellegrini M, Manini F, Ko MSH, Nagaraja R, Forabosco A, Schlessinger D. 2005. Mouse ovary developmental RNA and protein markers from gene expression profiling. *Dev Biol* **279:** (2) 271.
Imin N, Nizamidin M, Daniher D, Nolan KE, Rose RJ, Rolfe BG. 2005. Proteomic analysis of somatic embryogenesis in *Medicago truncatula*. Explant cultures grown under 6-benzylaminopurine and 1-napthaleneacetic acid treatments. *Plant Physiol* **137:** (4) 1250.
Jeong JA, Hong SH, Gang EJ, Ahn C, Hwang SH, Yang IH, Han H, Kim H. 2005. Differential gene expression profiling of human

Copyright © 2006 John Wiley & Sons, Ltd. 

umbilical cord blood-derived mesenchymal stem cells by DNA microarray. *Stem Cells* **23:** (4) 584.
Jordan KC, Hatfield SD, Tworoger M, Ward EJ, Fischer KA, Bowers S, Ruohola-Baker H. 2005. Genome wide analysis of transcript levels after perturbation of the EGFR pathway in the *Drosophila* ovary. *Dev Dyn* **232:** (3) 709.
Karoly ED, Schmid JE, Hunter ES. 2005. Ontogeny of transcription profiles during mouse early craniofacial development. *Reprod Toxicol* **19:** (3) 339.
Lai HS, Chen Y, Lin WH, Chen CN, Wu HC, Chang CJ, Lee PH, Chang KJ, Chen WJ. 2005. Quantitative gene expression analysis by cDNA microarray during liver regeneration after partial hepatectomy in rats. *Surg Today* **35:** (5) 396.
Lippert D, Zhuang J, Ralph S, Ellis DE, Gilbert M, Olafson R, Ritland K, Ellis B, Douglas CJ, Bohlmann J. 2005. Proteome analysis of early somatic embryogenesis in *Picea glauca*. *Proteomics* **5:** (2) 461.
Mariadason JM, Nicholas C, L'Italien KE, Zhuang M, Smartt HJM, Heerdt BG, Yang WC, Corner GA, Wilson AJ, Klampfer L *et al.* 2005. Gene expression profiling of intestinal epithelial cell maturation along the crypt-villus axis. *Gastroenterology* **128:** (4) 1081.
Maurer MH, Feldmann RE, Bromme JO, Kalenka A. 2005. Comparison of statistical approaches for the analysis of proteome expression data of differentiating neural stem cells. *J Proteome Res* **4:** (1) 96.
Nagano K, Taoka M, Yamauchi Y, Itagaki C, Shinkawa T, Nunomura K, Okamura N, Takahashi N, Izumi T, Isobe T. 2005. Large-scale identification of proteins expressed in mouse embryonic stem cells. *Proteomics* **5:** (5) 1346.
Park YK, Franklin JL, Settle SH, Levy SE, Chung E, Jeyakumar LH, Shyr Y, Washington MK, Whitehead RH, Aronow BJ *et al.* 2005. Gene expression profile analysis of mouse colon embryonic development. *Genesis* **41:** (1) 1.
Pollet N, Muncke N, Verbeek B, Li Y, Fenger U, Delius H, Niehrs C. 2005. An atlas of differential gene expression during early *Xenopus* embryogenesis. *Mech Dev* **122:** (3) 365.
Prowse ABJ, McQuade LR, Bryant KJ, Van Dyk DD, Tuch BE, Gray PP. 2005. A proteome analysis of conditioned media from human neonatal fibroblasts used in the maintenance of human embryonic stem cells. *Proteomics* **5:** (4) 978.
Shimada T, Fuiii H, Endo T, Yazaki J, Kishimoto N, Shimbo K, Kikuchi S, Omura M. 2005. Toward comprehensive expression profiling by microarray analysis in citrus: Monitoring the expression profiles of 2213 genes during fruit development. *Plant Sci* **168:** (5) 1383.
Sonnichsen B, Koski LB, Walsh A, Marschall P, Neumann B, Brehm M, Alleaume AM, Artelt J, Bettencourt P, Cassin E *et al.* 2005. Full-genome RNAi profiling of early embryogenesis in *Caenorhabditis elegans*. *Nature* **434:** (7032) 462.
Uemura O, Okada Y, Ando H, Guedj M, Higashijima S, Shimazaki T, Chino N, Okano H, Okamoto H. 2005. Comparative functional genomics revealed conservation and diversification of three enhancers of the *isl1* gene for motor and sensory neuron-specific expression. *Dev Biol* **278:** (2) 587.
Ushizawa K, Takahashi T, Kaneyama K, Tokunaga T, Tsunoda Y, Hashizume K. 2005. Gene expression profiles of bovine trophoblastic cell line (BT-1) analyzed by a custon cDNA microarray. *J Reprod Dev* **51:** (2) 211.
Vensel WH, Tanaka CK, Cai N, Wong JH, Buchanan BB, Hurkman WJ. 2005. Developmental changes in the metabolic protein profiles of wheat endosperm. *Proteomics* **5:** (6) 1594.
Voigt J, Chen JA, Gilchrist M, Amaya E, Papalopulu N. 2005. Expression cloning screening of a unique and full-length set of cDNA clones is an efficient method for identifying genes involved in *Xenopus* neurogenesis. *Mech Dev* **122:** (3) 289.
Wei CL, Miura T, Robson P, Lim SK, Xu XQ, Lee MYC, Gupta S, Stanton L, Luo YQ, Schmitt J *et al.* 2005. Transcriptome profiling of human and murine ESCs identifies divergent paths required to maintain the stem cell state. *Stem Cells* **23:** (2) 166.
Yoshida KT, Endo M, Nakazono M, Fukuda H, Demura T, Tsuchiya T, Watanabe M. 2005. cDNA microarray analysis of gene expression changes during pollination, pollen-tube elongation, fertilization, and early embryogenesis in rice pistils. *Sex Plant Reprod* **17:** (6) 269.
Zhao CF, Wang JQ, Cao ML, Zhao K, Shao JM, Lei TT, Yin JN, Hill GG, Xu NZ, Liu SQ. 2005. Proteomic changes in rice leaves during development of field-grown rice plants. *Proteomics* **5:** (4) 961.
Zhong JF, Zhao Y, Sutton S, Su A, Zhan YX, Zhu LJ, Yan CL, Gallaher T, Johnston PB, Anderson WF *et al.* 2005. Gene expression profile of murine long-term reconstituting *vs.* short-term reconstituting hematopoietic stem cells. *Proc Natl Acad Sci U S A* **102:** (7) 2448.

### I5 Technological advances

Aivado M, Spentzos D, Alterovitz G, Otu HH, Grall F, Giagounidis AAN, Wells M, Cho JY, Germing U, Czibere A, Prall WC, Porter C, Ramoni MF, Libermann TA. 2005. Optimization and evaluation of surface-enhanced laser desorption/ionization time-of-flight mass spectrometry (SELDI-TOF MS) with reversed-phase protein arrays for protein profiling. *Clin Chem Lab Med* **43:** (2) 133.
Almeida JS, Stanislaus R, Krug E, Arthur JM. 2005. Normalization and analysis of residual variation in two-dimensional gel electrophoresis for quantitative differential proteomics. *Proteomics* **5:** (5) 1242.
Almeida JS, McKillen DJ, Chen YA, Gross PS, Chapman RW, Warr G. 2005. Design and calibration of microarrays as universal transcriptomic environmental biosensors. *Comp Funct Genom* **6:** (3) 132.
Angenendt P, Lehrach H, Kreutzberger J, Glokler J. 2005. Subnanoliter enzymatic assays on microarrays. *Proteomics* **5:** (2) 420.
Baczek T, Wiczling P, Marszall M, Vander Heyden Y, Kaliszan R. 2005. Prediction of peptide retention at different HPLC conditions from multiple linear regression models. *J Proteome Res* **4:** (2) 555.
Ballell L, Alink KJ, Slijper M, Versluis C, Liskamp RMJ, Pieters RJ. 2005. A new chemical probe for proteomics of carbohydrate-binding proteins. *Chembiochem* **6:** (2) 291.
Calleri E, Temporini C, Perani E, De Palma A, Lubda D, Mellerio G, Sala A, Galliano M, Caccialanza G, Massolini G. 2005. Trypsin-based monolithic bioreactor coupled on-line with LC/MS/MS system for protein digestion and variant identification in standard solutions and serum samples. *J Proteome Res* **4:** (2) 481.
Cha T, Guo A, Zhu XY. 2005. Enzymatic activity on a chip: The critical role of protein orientation. *Proteomics* **5:** (2) 416.
Chen HS, Rejtar T, Andreev V, Moskovets E, Karger BL. 2005. High-speed, high-resolution monolithic capillary LC-MALDI MS using an off-line continuous deposition interface for proteomic analysis. *Anal Chem* **77:** (8) 2323.
Cockrill SL, Foster KL, Wildsmith J, Goodrich AR, Dapron JG, Hassell TC, Kappel WK, Scott GBI. 2005. Efficient micro-recovery and guanidination of peptides directly from MALDI target spots. *Biotechniques* **38:** (2) 301.
Cramer R, Corless S. 2005. Liquid ultraviolet matrix-assisted laser desorption/ionization mass spectrometry for automated proteomic analysis. *Proteomics* **5:** (2) 360.
Doherty MK, Whitehead C, McCormack H, Gaskell SJ, Beynon RJ. 2005. Proteome dynamics in complex organisms: Using stable isotopes to monitor individual protein turnover rates. *Proteomics* **5:** (2) 522.
Dong SY, Ma HM, Duan XJ, Chen XQ, Li J. 2005. Detection of local polarity of α-lactalbumin by *n*-terminal specific labeling with a new tailor-made fluorescent probe. *J Proteome Res* **4:** (1) 161.
Eriksson J, Fenyo D. 2005. Protein identification in complex mixtures. *J Proteome Res* **4:** (2) 387.
Ferguson RE, Carroll HP, Harris A, Maher ER, Selby PJ, Banks RE. 2005. Housekeeping proteins: A preliminary study illustrating some limitations as useful references in protein expression studies. *Proteomics* **5:** (2) 566.
Fortier MH, Bonneil E, Goodley P, Thibault P. 2005. Integrated microfluidic device for mass spectrometry-based proteomics and its application to biomarker discovery programs. *Anal Chem* **77:** (6) 1631.
Godovac-Zimmermann J, Kleiner O, Brown LR, Drukier AK. 2005. Perspectives in spicing up proteomics with splicing. *Proteomics* **5:** (3) 699.
Goldberg D, Sutton-Smith M, Paulson J, Dell A. 2005. Automatic annotation of matrix-assisted laser desorption/ionization *N*-glycan spectra. *Proteomics* **5:** (4) 865.
Gu X, Wang Y, Zhang XM. 2005. Large-bore particle-entrapped monolithic precolumns prepared by a sol-gel method for on-line peptides trapping and preconcentration in multidimensional liquid chromatography system for proteome analysis. *J Chromatogr A* **1072:** (2) 223.
Guerrier L, Lomas L, Boschetti E. 2005. A simplified monobuffer multidimensional chromatography for high-throughput proteome fractionation. *J Chromatogr A* **1073:** (1-2) 25.

Copyright © 2006 John Wiley & Sons, Ltd. 

Hagman C, Ramstrom M, Jansson M, James P, Hakansson P, Bergquist J. 2005. Reproducibility of tryptic digestion investigated by quantitative Fourier transform ion cyclotron resonance mass spectrometry. *J Proteome Res* **4:** (2) 394.
Harbig J, Sprinkle R, Enkemann SA. 2005. A sequence-based identification of the genes detected by probesets on the Affymetrix U133 plus 2.0 array. *Nucleic Acids Res* **33:** (3) e31.
Heller M, Michel PE, Morier P, Crettaz D, Wenz C, Tissot JD, Reymond F, Rossier JS. 2005. Two-stage Off-Gel™ isoelectric focusing: Protein followed by peptide fractionation and application to proteome analysis of human plasma. *Electrophoresis* **26:** (6) 1174.
Hogan JM, Pitteri SJ, Chrisman PA, McLuckey SA. 2005. Complementary structural information from a tryptic *N*-linked glycopeptide *via* electron transfer ion/ion reactions and collision-induced dissociation. *J Proteome Res* **4:** (2) 628.
Hsu JL, Huang SY, Shiea JT, Huang WY, Chen SH. 2005. Beyond quantitative proteomics: Signal enhancement of the $a_1$ ion as a mass tag for peptide sequencing using dimethyl labeling. *J Proteome Res* **4:** (1) 101.
Ishihama Y. 2005. Proteomic LC-MS systems using nanoscale liquid chromatography with tandem mass spectrometry. *J Chromatogr A* **1067:** (1-2) 73.
Juan HF, Chang SC, Huang HC, Chen ST. 2005. A new application of microwave technology to proteomics. *Proteomics* **5:** (4) 840.
Junttila MR, Saarinen S, Schmidt T, Kast J, Westermarck J. 2005. Single-step Strep-Tag® purification for the isolation and identification of protein complexes from mammalian cells. *Proteomics* **5:** (5) 1199.
Kaliszan R, Baczek T, Cimochowska A, Juszczyk P, Wisniewska K, Grzonka Z. 2005. Prediction of high-performance liquid chromatography retention of peptides with the use of quantitative structure-retention relationships. *Proteomics* **5:** (2) 409.
Kamoda S, Nakano M, Ishikawa R, Suzuki S, Kakehi K. 2005. Rapid and sensitive screening of *N*-glycans as 9-fluorenylmethyl derivatives by high-performance liquid chromatography: A method which can recover free oligosaccharides after analysis. *J Proteome Res* **4:** (1) 146.
Karlsson H, Leanderson P, Tagesson C, Lindahl M. 2005. Lipoproteomics I: Mapping of proteins in low-density lipoprotein using two-dimensional gel electrophoresis and mass spectrometry. *Proteomics* **5:** (2) 551.
Kawakami T, Tateishi K, Yamano Y, Ishikawa T, Kuroki K, Nishimura T. 2005. Protein identification from product ion spectra of peptides validated by correlation between measured and predicted elution times in liquid chromatography/mass spectrometry. *Proteomics* **5:** (4) 856.
Kendziorski C, Irizarry RA, Chen KS, Haag JD, Gould MN. 2005. On the utility of pooling biological samples in microarray experiments. *Proc Natl Acad Sci U S A* **102:** (12) 4252.
Kirkpatrick DS, Gerber SA, Gygi SP. 2005. The absolute quantification strategy: A general procedure for the quantification of proteins and post-translational modifications. *Methods* **35:** (3) 265.
Koeniger SL, Valentine SJ, Myung S, Plasencia M, Lee YJ, Clemmer DE. 2005. Development of field modulation in a split-field drift tube for high-throughput multidimensional separations. *J Proteome Res* **4:** (1) 25.
Lanne B, Panfilov O. 2005. Protein staining influences the quality of mass spectra obtained by peptide mass fingerprinting after separation on 2-D gels. A comparison of staining with Coomassie Brilliant Blue and Sypro Ruby. *J Proteome Res* **4:** (1) 175.
Levander F, James P. 2005. Automated protein identification by the combination of MALDI MS and MS/MS spectra from different instruments. *J Proteome Res* **4:** (1) 71.
Li XP, Wilm M, Franz T. 2005. Silicone/graphite coating for on-target desalting and improved peptide mapping performance of matrix-assisted laser desorption/ionization-mass spectrometry targets in proteomic experiments. *Proteomics* **5:** (6) 1460.
Liang Z, Zhang LH, Duan JC, Yan C, Zhang WB, Zhang YK. 2005. On-line concentration of proteins in pressurized capillary electrochromatography coupled with electrospray ionization-mass spectrometry. *Electrophoresis* **26:** (7-8) 1398.
Lippman Z, Gendrel AV, Colot V, Martienssen R. 2005. Profiling DNA methylation patterns using genomic tiling microarrays. *Nat Methods* **2:** (3) 219.
Markillie LM, Lin CT, Adkins JN, Auberry DL, Hill EA, Hooker BS, Moore PA, Moore RJ, Shi L, Wiley HS *et al*. 2005. Simple protein complex purification and identification method for high-throughput mapping of protein interaction networks. *J Proteome Res* **4:** (2) 268.
McCarthy FM, Burgess SC, Van den Berg BHJ, Koter MD, Pharr GT. 2005. Differential detergent fractionation for non-electrophoretic eukaryote cell proteomics. *J Proteome Res* **4:** (2) 316.
Mirgorodskaya E, Braeuer C, Fucini P, Lehrach H, Gobom J. 2005. Nanoflow liquid chromatography coupled to matrix-assisted laser desorption/ionization mass spectrometry: Sample preparation, data analysis, and application to the analysis of complex peptide mixtures. *Proteomics* **5:** (2) 399.
Molloy MP, Donohoe S, Brzezinski EE, Kilby GW, Stevenson TI, Baker JD, Goodlett DR, Gage DA. 2005. Large-scale evaluation of quantitative reproducibility and proteome coverage using acid cleavable isotope coded affinity tag mass spectrometry for proteomic profiling. *Proteomics* **5:** (5) 1204.
Nakayashiki H, Hanada S, Quoc NB, Kadotani N, Tosa Y, Mayama S. 2005. RNA silencing as a tool for exploring gene function in ascomycete fungi. *Fungal Genet Biol* **42:** (4) 275.
Pashkova A, Chen HS, Rejtar T, Zang X, Giese R, Andreev V, Moskovets E, Karger BL. 2005. Coumarin tags for analysis of peptides by MALDI-TOF MS and MS/MS. 2. Alexa Fluor 350 tag for increased peptide and protein identification by LC-MALDI-TOF/TOF MS. *Anal Chem* **77:** (7) 2085.
Patel OV, Suchyta SP, Sipkovsky SS, Yao JB, Ireland JJ, Coussens PM, Smith GW. 2005. Validation and application of a high fidelity mRNA linear amplification procedure for profiling gene expression. *Vet Immunol Immunopathol* **105:** (3-4) 331.
Qiu RQ, Regnier FE. 2005. Use of multidimensional lectin affinity chromatography in differential glycoproteomics. *Anal Chem* **77:** (9) 2802.
Riter LS, Hodge BD, Gooding KM, Julian RK. 2005. Use of lysozyme as a standard for evaluating the effectiveness of a proteomics process. *J Proteome Res* **4:** (1) 153.
Rosa GJM, Steibel JP, Tempelman RJ. 2005. Reassessing design and analysis of two-colour microarray experiments using mixed effects models. *Comp Funct Genom* **6:** (3) 123.
Salek M, Di Bartolo V, Cittaro D, Borsotti D, Wei JH, Acuto O, Rappsilber J, Lehmann WD. 2005. Sequence tag scanning: A new explorative strategy for recognition of unexpected protein alterations by nanoelectrospray ionization-tandem mass spectrometry. *Proteomics* **5:** (3) 667.
Sanz-Nebot V, Balaguer E, Benavente F, Barbosa J. 2005. Comparison of sheathless and sheath-flow electrospray interfaces for the capillary electrophoresis-electrospray ionization-mass spectrometry analysis of peptides. *Electrophoresis* **26:** (7-8) 1457.
Schneider LV, Hall MR. 2005. Stable isotope method for high-precision proteomics. *Drug Discov Today* **10:** (5) 353.
Searle BC, Dasari S, Wilmarth PA, Turner M, Reddy AP, David LL, Nagalla SR. 2005. Identification of protein modifications using MS/MS *de novo* sequencing and the OpenSea alignment algorithm. *J Proteome Res* **4:** (2) 546.
Shieh IF, Lee CY, Shiea J. 2005. Eliminating the interferences from TRIS buffer and SDS in protein analysis by fused-droplet electrospray ionization mass spectrometry. *J Proteome Res* **4:** (2) 606.
Shui WQ, Liu YK, Fan HZ, Bao HM, Liang SF, Yang PY, Chen X. 2005. Enhancing TOF/TOF-based *de novo* sequencing capability for high throughput protein identification with amino acid-coded mass tagging. *J Proteome Res* **4:** (1) 83.
Silva JC, Denny R, Dorschel CA, Gorenstein M, Kass IJ, Li GZ, McKenna T, Nold MJ, Richardson K, Young P, Geromanos S. 2005. Quantitative proteomic analysis by accurate mass retention time pairs. *Anal Chem* **77:** (7) 2187.
Snijders APL, De Vos MGJ, Wright PC. 2005. Novel approach for peptide quantitation and sequencing based on $^{15}N$ and $^{13}C$ metabolic labeling. *J Proteome Res* **4:** (2) 578.
Stackebrandt E, Pauker O, Erhard M. 2005. Grouping myxococci (*Corallococcus*) strains by matrix-assisted laser desorption ionization time-of-flight (MALDI TOF) mass spectrometry: Comparison with gene sequence phylogenies. *Current Microbiol* **50:** (2) 71.
Stevens JR, Doerge RW. 2005. Meta-analysis combines Affymetrix microarray results across laboratories. *Comp Funct Genom* **6:** (3) 116.
Stutz H. 2005. Advances in the analysis of proteins and peptides by capillary electrophoresis with matrix-assisted laser desorption/ionization and electrospray-mass spectrometry detection (Review). *Electrophoresis* **26:** (7-8) 1254.

Copyright © 2006 John Wiley & Sons, Ltd. 

Szpunar J. 2005. Advances in analytical methodology for bioinorganic speciation analysis; metallomics, metalloproteomics and hetero-atom-tagged proteomics and metabolomics. *Analyst* **130:** (4) 442.
Tempez A, Ugarov M, Egan T, Schultz JA, Novikov A, Della-Negra S, Lebeyec Y, Pautrat M, Caroff M, Smentkowski VS, Wang HY, Jackson SN, Woods AS. 2005. Matrix implanted laser desorption ionization (MILDI) combined with ion mobility-mass spectrometry for bio-surface analysis. *J Proteome Res* **4:** (2) 540.
Venne K, Bonneil E, Eng K, Thibault P. 2005. Improvement in peptide detection for proteomics analyses using nanoLC-MS and high-field asymmetry waveform ion mobility mass spectrometry. *Anal Chem* **77:** (7) 2176.
Wang YJ, Balgley BM, Rudnick PA, Lee CS. 2005. Effects of chromatography conditions on intact protein separations for top-down proteomics. *J Chromatogr A* **1073:** (1-2) 35.
Wilson ID, Nicholson JK, Castro-Perez J, Granger JH, Johnson KA, Smith BW, Plumb RS. 2005. High resolution "ultra performance" liquid chromatography coupled to oa-TOF mass spectrometry as a tool for differential metabolic pathway profiling in functional genomic studies. *J Proteome Res* **4:** (2) 591.
Wingren C, Steinhauer C, Ingvarsson J, Persson E, Larsson K, Borrebaeck CAK. 2005. Microarrays based on affinity-tagged single-chain Fv antibodies: Sensitive detection of analyte in complex proteomes. *Proteomics* **5:** (5) 1281.
Xu M, Geer LY, Bryant SH, Roth JS, Kowalak JA, Maynard DM, Markey SP. 2005. Assessing data quality of peptide mass spectra obtained by quadrupole ion trap mass spectrometry. *J Proteome Res* **4:** (2) 300.
Yoshitani N, Satou K, Saito K, Suzuki S, Hatanaka H, Seki M, Shinozaki K, Hirota H, Yokoyama S. 2005. A structure-based strategy for discovery of small ligands binding to functionally unknown proteins; Combination of *in silico* screening and surface plasmon resonance measurements. *Proteomics* **5:** (6) 1472.
Zhang JH, Finney RP, Clifford RJ, Derr LK, Buetow KH. 2005. Detecting false expression signals in high-density oligonucleotide arrays by an *in silico* approach. *Genomics* **85:** (3) 297.
Zhao YP, Lin YH. 2005. A proteomic tool for protein identification from tandem mass spectral data. *Proteomics* **5:** (4) 853.
Zhou F, Johnston MV. 2005. Protein profiling by capillary isoelectric focusing, reversed-phase liquid chromatography, and mass spectrometry. *Electrophoresis* **26:** (7-8) 1383.

### I6 Bioinformatics

Abul O, Alhajj R, Polat F, Barker K. 2005. Finding differentially expressed genes for pattern generation. *Bioinformatics* **21:** (4) 445.
Aitken S, Korf R, Webber B, Bard J. 2005. COBrA: A bio-ontology editor. *Bioinformatics* **21:** (6) 825.
Arnesano F, Banci L, Bertini I, Martinelli M. 2005. Ortholog search of proteins involved in copper delivery to cytochrome *c* oxidase and functional analysis of paralogs and gene neighbors by genomic context. *J Proteome Res* **4:** (1) 63.
Asyali MH, Alci M. 2005. Reliability analysis of microarray data using fuzzy c-means and normal mixture modeling based classification methods. *Bioinformatics* **21:** (5) 644.
Bakewell DJ, Wit E. 2005. Weighted analysis of microarray gene expression using maximum-likelihood. *Bioinformatics* **21:** (6) 723.
Balasubramaniyan R, Hullermeier E, Weskamp N, Kamper J. 2005. Clustering of gene expression data using a local shape-based similarity measure. *Bioinformatics* **21:** (7) 1069.
Barash Y, Elidan G, Kaplan T, Friedman N. 2005. CIS: Compound importance sampling method for protein-DNA binding site *p*-value estimation. *Bioinformatics* **21:** (5) 596.
Bartels D, Kespohl S, Albaum S, Druke T, Goesmann A, Herold J, Kaiser O, Puhler A, Pfeiffer F, Raddatz G *et al*. 2005. BACCardI - A tool for the validation of genomic assemblies, assisting genome finishing and intergenome comparison. *Bioinformatics* **21:** (7) 853.
Betancourt L, Gil J, Besada V, Gonzalez LJ, Fernandez-de-Cossio J, Garcia L, Pajon R, Sanchez A, Alvarez F, Padron G. 2005. SCAPE: A new tool for the Selective CApture of PEptides in protein identification. *J Proteome Res* **4:** (2) 491.
Beyer A, Wilhelm T. 2005. Dynamic simulation of protein complex formation on a genomic scale. *Bioinformatics* **21:** (8) 1610.
Beynon RJ. 2005. A simple tool for drawing proteolytic peptide maps. *Bioinformatics* **21:** (5) 674.
Bilke S, Chen QR, Whiteford CC, Khan J. 2005. Detection of low level genomic alterations by comparative genomic hybridization based on cDNA micro-arrays. *Bioinformatics* **21:** (7) 1138.
Boby T, Patch AM, Aves SJ. 2005. TRbase: A database relating tandem repeats to disease genes for the human genome. *Bioinformatics* **21:** (6) 811.
Boissy G, Wojcik J. 2005. Bioinformatic analysis of proteomic interactions: Combining quantity and quality. *Biofutur* **252:** 32.
Bokhari SH, Sauer JR. 2005. A parallel graph decomposition algorithm for DNA sequencing with nanopores. *Bioinformatics* **21:** (7) 889.
Bolshakova N, Azuaje F, Cunningham P. 2005. An integrated tool for microarray data clustering and cluster validity assessment. *Bioinformatics* **21:** (4) 451.
Bozdech Z, Ginsburg H. 2005. Data mining of the transcriptome of *Plasmodium falciparum*: The pentose phosphate pathway and ancillary processes. *Malaria J* **4:** (18) 17.
Bradford JR, Westhead DR. 2005. Improved prediction of protein-protein binding sites using a support vector machines approach. *Bioinformatics* **21:** (8) 1487.
Buness A, Huber W, Steiner K, Sultmann H, Poustka A. 2005. arrayMagic: Two-colour cDNA microarray quality control and preprocessing. *Bioinformatics* **21:** (4) 554.
Burden S, Lin YX, Zhang R. 2005. Improving promoter prediction for the NNPP2.2 algorithm: A case study using *Escherichia coli* DNA sequences. *Bioinformatics* **21:** (5) 601.
Campagna D, Romualdi C, Vitulo N, Del Favero M, Lexa M, Cannata N, Valle G. 2005. RAP: A new computer program for *de novo* identification of repeated sequences in whole genomes. *Bioinformatics* **21:** (5) 582.
Caraux G, Pinloche S. 2005. PermutMatrix: A graphical environment to arrange gene expression profiles in optimal linear order. *Bioinformatics* **21:** (7) 1280.
Charif D, Thioulouse J, Lobry JR, Perriere G. 2005. Online synonymous codon usage analyses with the ade4 and seqinR packages. *Bioinformatics* **21:** (4) 545.
Chen TM, Lu CC, Li WH. 2005. Prediction of splice sites with dependency graphs and their expanded bayesian networks. *Bioinformatics* **21:** (4) 471.
Chen Y, Xu D. 2005. Understanding protein dispensability through machine-learning analysis of high-throughput data. *Bioinformatics* **21:** (5) 575.
Costa LD. 2005. Biological sequence analysis through the one-dimensional percolation transform and its enhanced version. *Bioinformatics* **21:** (5) 608.
Cui QH, Liu B, Jiang TX, Ma SD. 2005. Characterizing the dynamic connectivity between genes by variable parameter regression and Kalman filtering based on temporal gene expression data. *Bioinformatics* **21:** (8) 1538.
Dalmasso C, Broet P, Moreau T. 2005. A simple procedure for estimating the false discovery rate. *Bioinformatics* **21:** (5) 660.
De Givry S, Bouchez M, Chabrier P, Milan D, Schiex T. 2005. $CAR_H{}^TAGene$: Multipopulation integrated genetic and radiation hybrid mapping. *Bioinformatics* **21:** (8) 1703.
Degroeve S, Saeys Y, De Baets B, Rouze P, Van de Peer Y. 2005. SpliceMachine: Predicting splice sites from high-dimensional local context representations. *Bioinformatics* **21:** (8) 1332.
Dehnert M, Helm WE, Hutt MT. 2005. Information theory reveals large-scale synchronisation of statistical correlations in eukaryote genomes. *Gene* **345:** (1) 81.
Delmar P, Robin S, Daudin JJ. 2005. VarMixt: Efficient variance modelling for the differential analysis of replicated gene expression data. *Bioinformatics* **21:** (4) 502.
Dinel S, Bolduc C, Belleau P, Boivin A, Yoshioka M, Calvo E, Piedboeuf B, Snyder EE, Labrie F, St-Amand J. 2005. Reproducibility, bioinformatic analysis and power of the SAGE method to evaluate changes in transcriptome. *Nucleic Acids Res* **33:** (3) e26.
Do CB, Mahabhashyam MSP, Brudno M, Batzoglou S. 2005. ProbCons: Probabilistic consistency-based multiple sequence alignment. *Genome Res* **15:** (2) 330.
Dobrokhotov PB, Goutte C, Veuthey AL, Gaussier E. 2005. Assisting

Copyright © 2006 John Wiley & Sons, Ltd. 

medical annotation in Swiss-Prot using statistical classifiers. *Int J Med Inf* **74:** (2-4) 317.
Eckel JE, Gennings C, Therneau TM, Burgoon LD, Boverhof DR, Zacharewski TR. 2005. Normalization of two-channel microarray experiments: A semiparametric approach. *Bioinformatics* **21:** (7) 1078.
Eddy SR. 2005. A model of the statistical power of comparative genome sequence analysis. *PLoS Biol* **3:** (1) e10.
Felder M, Szafranski K, Lehmann R, Eichinger L, Noegel AA, Platzer M, Glockner G. 2005. DictyMOLD - A *Dictyostelium discoideum* genome browser database. *Bioinformatics* **21:** (5) 696.
Fort G, Lambert-Lacroix S. 2005. Classification using partial least squares with penalized logistic regression. *Bioinformatics* **21:** (7) 1104.
Friedel CC, Jahn KHV, Sommer S, Rudd S, Mewes HW, Tetko IV. 2005. Support vector machines for separation of mixed plant-pathogen EST collections based on codon usage. *Bioinformatics* **21:** (8) 1383.
Futschik ME, Crompton T. 2005. OLIN: Optimized normalization, visualization and quality testing of two-channel microarray data. *Bioinformatics* **21:** (8) 1724.
Gardy JL, Laird MR, Chen F, Rey S, Walsh CJ, Ester M, Brinkman FSL. 2005. PSORTb v.2.0: Expanded prediction of bacterial protein subcellular localization and insights gained from comparative proteome analysis. *Bioinformatics* **21:** (5) 617.
Gough J. 2005. Convergent evolution of domain architectures (is rare). *Bioinformatics* **21:** (8) 1464.
Graham J, McNeney B, Seillier-Moiseiwitsch F. 2005. Stepwise detection of recombination breakpoints in sequence alignments. *Bioinformatics* **21:** (5) 589.
Gromiha MM, Suwa M. 2005. A simple statistical method for discriminating outer membrane proteins with better accuracy. *Bioinformatics* **21:** (7) 961.
Gu X, Huang W, Xu DP, Zhang HM. 2005. GeneContent: Software for whole-genome phylogenetic analysis. *Bioinformatics* **21:** (8) 1713.
Guan Z, Zhao HY. 2005. A semiparametric approach for marker gene selection based on gene expression data. *Bioinformatics* **21:** (4) 529.
Guthke R, Moller U, Hoffmann M, Thies F, Topfer S. 2005. Dynamic network reconstruction from gene expression data applied to immune response during bacterial infection. *Bioinformatics* **21:** (8) 1626.
Hansen BT, Davey SW, Ham AJL, Liebler DC. 2005. P-Mod: An algorithm and software to map modifications to peptide sequences using tandem MS data. *J Proteome Res* **4:** (2) 358.
Huang YC, Pumphrey J, Gingle AR. 2005. ESTminer: A Web interface for mining EST contig and cluster databases. *Bioinformatics* **21:** (5) 669.
Itoh M, Goto S, Akutsu T, Kanehisa M. 2005. Fast and accurate database homology search using upper bounds of local alignment scores. *Bioinformatics* **21:** (7) 912.
Jacob E, Sasikumar R, Nair KNR. 2005. A fuzzy guided genetic algorithm for operon prediction. *Bioinformatics* **21:** (8) 1403.
Ji LP, Tan KL. 2005. Identifying time-lagged gene clusters using gene expression data. *Bioinformatics* **21:** (4) 509.
Ji Y, Tsui KW, Kim KM. 2005. A novel means of using gene clusters in a two-step empirical Bayes method for predicting classes of samples. *Bioinformatics* **21:** (7) 1055.
Jungo F, Bairoch A. 2005. Tox-Prot, the toxin protein annotation program of the Swiss-Prot protein knowledgebase. *Toxicon* **45:** (3) 293.
Kann MG, Thiessen PA, Panchenko AR, Schaffer AA, Altschul SF, Bryant SH. 2005. A structure-based method for protein sequence alignment. *Bioinformatics* **21:** (8) 1451.
Kaplinski L, Andreson R, Puurand T, Remm M. 2005. MultiPLX: Automatic grouping and evaluation of PCR primers. *Bioinformatics* **21:** (8) 1701.
Kasturi J, Acharya R. 2005. Clustering of diverse genomic data using information fusion. *Bioinformatics* **21:** (4) 423.
Kemmeren P, Kockelkorn TTJP, Bijma T, Donders R, Holstege FCP. 2005. Predicting gene function through systematic analysis and quality assessment of high-throughput data. *Bioinformatics* **21:** (8) 1644.
Kifer I, Sasson O, Linial M. 2005. Predicting fold novelty based on ProtoNet hierarchical classification. *Bioinformatics* **21:** (7) 1020.
Kim BS, Kim I, Lee S, Kim S, Rha SY, Chung HC. 2005. Statistical methods of translating microarray data into clinically relevant diagnostic information in colorectal cancer. *Bioinformatics* **21:** (4) 517.
Kim J, Seo J, Lee YS, Kim S. 2005. TFExplorer: Integrated analysis database for predicted transcription regulatory elements. *Bioinformatics* **21:** (4) 548.
Kim TH, Jeon YJ, Kim WY, Kim HS. 2005. HESAS: HERVs Expression and Structure Analysis System. *Bioinformatics* **21:** (8) 1699.
Kiryu H, Oshima T, Asai K. 2005. Extracting relations between promoter sequences and their strengths from microarray data. *Bioinformatics* **21:** (7) 1062.
Kisman D, Li M, Ma B, Wang L. 2005. tPatternHunter: Gapped, fast and sensitive translated homology search. *Bioinformatics* **21:** (4) 542.
Kloster M, Tang C, Wingreen NS. 2005. Finding regulatory modules through large-scale gene-expression data analysis. *Bioinformatics* **21:** (7) 1172.
Komura D, Nakamura H, Tsutsumi S, Aburatani H, Ihara S. 2005. Multidimensional support vector machines for visualization of gene expression data. *Bioinformatics* **21:** (4) 439.
Kopka J, Schauer N, Krueger S, Birkemeyer C, Usadel B, Bergmuller E, Dormann P, Weckwerth W, Gibon Y, Stitt M *et al.* 2005. GMD@CSB.DB: The Golm Metabolome Database. *Bioinformatics* **21:** (8) 1635.
Kreil DP, Russell RR. 2005. There is no silver bullet - A guide to low-level data transforms and normalisation methods for microarray data. *Brief Bioinform* **6:** (1) 86.
Li JB, Zhang M, Dutcher SK, Stormo GD. 2005. Procom: A web-based tool to compare multiple eukaryotic proteomes. *Bioinformatics* **21:** (8) 1693.
Li WX, Rehmeyer CJ, Staben C, Farman ML. 2005. TERMINUS-Telomeric End-Read Mining IN Unassembled Sequences. *Bioinformatics* **21:** (8) 1695.
Lin DY. 2005. An efficient Monte Carlo approach to assessing statistical significance in genomic studies. *Bioinformatics* **21:** (6) 781.
Lingjaerde OC, Baumbusch LO, Liestol K, Glad IK, Borresen-Dale AL. 2005. CGH-Explorer: A program for analysis of array-CGH data. *Bioinformatics* **21:** (6) 821.
Liu HQ, Han H, Li JY, Wong LS. 2005. DNAFSMiner: A web-based software toolbox to recognize two types of functional sites in DNA sequences. *Bioinformatics* **21:** (5) 671.
Lo SL, Cai CZ, Chen YZ, Chung MCM. 2005. Effect of training datasets on support vector machine prediction of protein-protein interactions. *Proteomics* **5:** (4) 876.
Makalowska I, Lin CF, Makalowski W. 2005. Overlapping genes in vertebrate genomics. *Comput Biol Chem* **29:** (1) 1.
Malde K, Coward E, Jonassen I. 2005. A graph based algorithm for generating EST consensus sequences. *Bioinformatics* **21:** (8) 1371.
Mikkelsen TS, Galagan JE, Mesirov JP. 2005. Improving genome annotations using phylogenetic profile anomaly detection. *Bioinformatics* **21:** (4) 464.
Miklos I, Ittzes P, Hein J. 2005. ParIS Genome Rearrangement server. *Bioinformatics* **21:** (6) 817.
Morgenstern B, Werner N, Prohaska SJ, Steinkamp R, Schneider I, Subramanian AR, Stadler PF, Weyer-Menkhoff J. 2005. Multiple sequence alignment with user-defined constraints at GOBICS. *Bioinformatics* **21:** (7) 1271.
Nelson RT, Grant D, Shoemaker RC. 2005. ESTminer: A suite of programs for gene and allele identification. *Bioinformatics* **21:** (5) 691.
Ng JKK, Liu WT. 2005. LabArray: Real-time imaging and analytical tool for microarrays. *Bioinformatics* **21:** (5) 689.
Nielsen HB, Gautier L, Knudsen S. 2005. Implementation of a gene expression index calculation method based on the PDNN model. *Bioinformatics* **21:** (5) 687.
Nordberg EK. 2005. YODA: Selecting signature oligonucleotides. *Bioinformatics* **21:** (8) 1365.
Nozaki Y, Bellgard M. 2005. Statistical evaluation and comparison of a pairwise alignment algorithm that a priori assigns the number of gaps rather than employing gap penalties. *Bioinformatics* **21:** (8) 1421.
Paar V, Pavin N, Rosandic M, Gluncic M, Basar I, Pezer R, Zinic SD. 2005. ColorHOR-novel graphical algorithm for fast scan of α satellite higher-order repeats and HOR annotation for GenBank sequence of human genome. *Bioinformatics* **21:** (7) 846.
Pal R, Datta A, Fornace AJ, Bittner ML, Dougherty ER. 2005. Boolean relationships among genes responsive to ionizing radiation in the NCI 60 ACDS. *Bioinformatics* **21:** (8) 1542.
Park YR, Park CH, Kim JH. 2005. GOChase: Correcting errors from

Copyright © 2006 John Wiley & Sons, Ltd. 

Gene Ontology-based annotations for gene products. *Bioinformatics* **21:** (6) 829.
Pinto FR, Cowart LA, Hannun YA, Rohrer B, Almeida JS. 2005. Local correlation of expression profiles with gene annotations-proof of concept for a general concilliatory method. *Bioinformatics* **21:** (7) 1037.
Pond SLK, Frost SDW, Muse SV. 2005. HyPhy: Hypothesis testing using phylogenies. *Bioinformatics* **21:** (5) 676.
Ponomarenko JV, Bourne PE, Shindyalov IN. 2005. Assigning new GO annotations to protein data bank sequences by combining structure and sequence homology. *Proteins* **58:** (4) 855.
Prigent V, Thierry JC, Poch O, Plewniak F. 2005. DbW: Automatic update of a functional family-specific multiple alignment. *Bioinformatics* **21:** (8) 1437.
Qian WJ, Liu T, Monroe ME, Strittmatter EF, Jacobs JM, Kangas LJ, Petritis K, Camp DG, Smith RD. 2005. Probability-based evaluation of peptide and protein identifications from tandem mass spectrometry and SEQUEST analysis: The human proteome. *J Proteome Res* **4:** (1) 53.
Rajagopalan D, Agarwal P. 2005. Inferring pathways from gene lists using a literature-derived network of biological relationships. *Bioinformatics* **21:** (6) 788.
Rimour S, Hill D, Militon C, Peyret P. 2005. GoArrays: Highly dynamic and efficient microarray probe design. *Bioinformatics* **21:** (7) 1094.
Robson B, Mushlin R. 2005. Genomic messaging system language including command extensions for clinical data categories. *J Proteome Res* **4:** (2) 275.
Robson B. 2005. Clinical and pharmacogenomic data mining: 3. Zeta Theory as a general tactic for clinical bioinformatics. *J Proteome Res* **4:** (2) 445.
Ronald J, Akey JM, Whittle J, Smith EN, Yvert G, Kruglyak L. 2005. Simultaneous genotyping, gene-expression measurement, and detection of allele-specific expression with oligonucleotide arrays. *Genome Res* **15:** (2) 284.
Sabatti C, Rohlin L, Lange K, Liao JC. 2005. Vocabulon: A dictionary model approach for reconstruction and localization of transcription factor binding sites. *Bioinformatics* **21:** (7) 922.
Sachdeva G, Kumar K, Jain P, Ramachandran S. 2005. SPAAN: A software program for prediction of adhesins and adhesin-like proteins using neural networks. *Bioinformatics* **21:** (4) 483.
Santoyo J, Vaquerizas JM, Dopazo J. 2005. Highly specific and accurate selection of siRNAs for high-throughput functional assays. *Bioinformatics* **21:** (8) 1376.
Sarachu M, Colet M. 2005. wEMBOSS: A web interface for EMBOSS. *Bioinformatics* **21:** (4) 540.
Sarkans U, Parkinson H, Lara GG, Oezcimen A, Sharma A, Abeygunawardena N, Contrino S, Holloway E, Rocca-Serra P, Mukherjee G *et al*. 2005. The ArrayExpress gene expression database: A software engineering and implementation perspective. *Bioinformatics* **21:** (8) 1495.
Sauer U, Preininger C, Hany-Schmatzberger R. 2005. Quick and simple: Quality control of microarray data. *Bioinformatics* **21:** (8) 1572.
Schafer J, Strimmer K. 2005. An empirical Bayes approach to inferring large-scale gene association networks. *Bioinformatics* **21:** (6) 754.
Schwager C, Blake J. 2005. Bloader - A batch loader application for MIAMExpress. *Bioinformatics* **21:** (8) 1727.
Soding J. 2005. Protein homology detection by HMM-HMM comparison. *Bioinformatics* **21:** (7) 951.
Statnikov A, Aliferis CF, Tsamardinos I, Hardin D, Levy S. 2005. A comprehensive evaluation of multicategory classification methods for microarray gene expression cancer diagnosis. *Bioinformatics* **21:** (5) 631.
Stothard P, Wishart DS. 2005. Circular genome visualization and exploration using CGView. *Bioinformatics* **21:** (4) 537.
Tabb DL, Narsimhan C, Strader MB, Hettich RL. 2005. DBDigger: Reorganized proteomic database identification that improves flexibility and speed. *Anal Chem* **77:** (8) 2464.
Taguchi Y, Oono Y. 2005. Relational patterns of gene expression *via* non-metric multidimensional scaling analysis. *Bioinformatics* **21:** (6) 730.
Troyanskaya OG. 2005. Putting microarrays in a context: Integrated analysis of diverse biological data. *Brief Bioinform* **6:** (1) 34.
Tsai CA, Lee TC, Ho IC, Yang UC, Chen CH, Chen JJ. 2005. Multi-class clustering and prediction in the analysis of microarray data. *Math Biosci* **193:** (1) 79.
Tsai CA, Wang SJ, Chen DT, Chen JJ. 2005. Sample size for gene expression microarray experiments. *Bioinformatics* **21:** (8) 1502.
Urbanczik R, Wagner C. 2005. An improved algorithm for stoichiometric network analysis: Theory and applications. *Bioinformatics* **21:** (7) 1203.
Van Walle I, Lasters I, Wyns L. 2005. SABmark - A benchmark for sequence alignment that covers the entire known fold space. *Bioinformatics* **21:** (7) 1267.
Via A, Zanzoni A, Helmer-Citterich M. 2005. Seq2Struct: A resource for establishing sequence-structure links. *Bioinformatics* **21:** (4) 551.
Vinciotti V, Khanin R, D'Alimonte D, Liu X, Cattini N, Hotchkiss G, Bucca G, De Jesus O, Rasaiyaah J, Smith CP *et al*. 2005. An experimental evaluation of a loop *versus* a reference design for two-channel microarrays. *Bioinformatics* **21:** (4) 492.
Wahl MB, Heinzmann U, Imai K. 2005. LongSAGE analysis revealed the presence of a large number of novel antisense genes in the mouse genome. *Bioinformatics* **21:** (8) 1389.
Wahl MB, Heinzmann U, Imai K. 2005. LongSAGE analysis significantly improves genome annotation: Identifications of novel genes and alternative transcripts in the mouse genome. *Bioinformatics* **21:** (8) 1393.
Wallace IM, Orla O, Higgins DG. 2005. Evaluation of iterative alignment algorithms for multiple alignment. *Bioinformatics* **21:** (8) 1408.
Wang YH, Makedon FS, Ford JC, Pearlman J. 2005. HykGene: A hybrid approach for selecting marker genes for phenotype classification using microarray gene expression data. *Bioinformatics* **21:** (8) 1530.
Westbrook J, Ito N, Nakamura H, Henrick K, Berman HM. 2005. PDBML: The representation of archival macromolecular structure data in XML. *Bioinformatics* **21:** (7) 988.
Wu BL. 2005. Differential gene expression detection using penalized linear regression models: The improved SAM statistics. *Bioinformatics* **21:** (8) 1565.
Xing B, Van Der Laan MJ. 2005. A statistical method for constructing transcriptional regulatory networks using gene expression and sequence data. *J Comput Biol* **12:** (2) 229.
Xu J, Gordon JI. 2005. MapLinker: A software tool that aids physical map-linked whole genome shotgun assembly. *Bioinformatics* **21:** (7) 1265.
Xu JF, Yang YN, Ott J. 2005. Survival analysis of microarray expression data by transformation models. *Comput Biol Chem* **29:** (2) 91.
Yamada T, Morishita S. 2005. Accelerated off-target search algorithm for siRNA. *Bioinformatics* **21:** (8) 1316.
Yan B, Pan C, Olman VN, Hettich RL, Xu Y. 2005. A graph-theoretic approach for the separation of b and y ions in tandem mass spectra. *Bioinformatics* **21:** (5) 563.
Yanai I, Benjamin H, Shmoish M, Califa-Caspi V, Shklar M, Ophir R, Bar-Even A, Horn-Saban S, Safran M, Domany E *et al*. 2005. Genome-wide midrange transcription profiles reveal expression level relationships in human tissue specification. *Bioinformatics* **21:** (5) 650.
Yandell M, Bailey AM, Misra S, Shu SQ, Wiel C, Evans-Holm M, Celniker SE, Rubin GM. 2005. A computational and experimental approach to validating annotations and gene predictions in the *Drosophila melanogaster* genome. *Proc Natl Acad Sci U S A* **102:** (5) 1566.
Yang YH, Xiao YY, Segal MR. 2005. Identifying differentially expressed genes from microarray experiments *via* statistic synthesis. *Bioinformatics* **21:** (7) 1084.
Yin P, Hartemink AJ. 2005. Theoretical and practical advances in genome halving. *Bioinformatics* **21:** (7) 869.
Zhao YP, Lin YH. 2005. The development of an algorithm for the mass spectral interpretation of phosphoproteins. *Proteomics* **5:** (4) 843.
Zheng XH, Lu F, Wang ZY, Hoover J, Mural R. 2005. Using shared genomic synteny and shared protein functions to enhance the identification of orthologous gene pairs. *Bioinformatics* **21:** (6) 703.
Zhou X, Mao KZ. 2005. LS Bound based gene selection for DNA microarray data. *Bioinformatics* **21:** (8) 1559.
Zhou Y, Mishra B. 2005. Quantifying the mechanisms for segmental duplications in mammalian genomes by statistical analysis and modeling. *Proc Natl Acad Sci U S A* **102:** (11) 4081.
Zhou YY, Young JA, Santrosyan A, Chen KS, Yan SF, Winzeler EA. 2005. *In silico* gene function prediction using ontology-based pattern identification. *Bioinformatics* **21:** (7) 1237.

Copyright © 2006 John Wiley & Sons, Ltd. 
